# Supplementary material for: Fumarate Hydratase Deletion in Pancreatic β Cells Leads to Progressive Diabetes
Source: Cell Rep. 2017 Sep 26;20(13):3135–48. doi: 10.1016/j.celrep.2017.08.093 (PMC5637167; doi:10.1016/j.celrep.2017.08.093)
Supplement: Document S1. Supplemental Experimental Procedures, Figures S1–S7, and Table S1 [file mmc1.pdf]

Cell Reports, Volume *20*

Supplemental Information

Fumarate Hydratase Deletion in Pancreatic β Cells Leads to Progressive Diabetes

Julie Adam, Reshma Ramracheya, Margarita V. Chibalina, Nicola Ternette, Alexander Hamilton, Andrei I. Tarasov, Quan Zhang, Eduardo Rebelato, Nils J.G. Rorsman, Rafael Martín-del-Río, Amy Lewis, Gizem Özkan, Hyun Woong Do, Peter Spégel, Kaori Saitoh, Keiko Kato, Kaori Igarashi, Benedikt M. Kessler, Christopher W. Pugh, Jorge Tamarit-Rodriguez, Hindrik Mulder, Anne Clark, Norma Frizzell, Tomoyoshi Soga, Frances M. Ashcroft, Andrew Silver, Patrick J. Pollard, and Patrik Rorsman

Supplemental Index

Supplemental Experimental Procedures
Supplemental References
Supplemental Table and Figure Legends
Supplemental Figure S1. Introduction of full length (FH) and cytoplasmic (FH^{cyt}) human fumarate hydratase rescues dysregulated metabolism in Fh1βKO islets.
Supplemental Figure S2. Glucose stimulated insulin secretion is blunted in Fh1βKO islets
Supplemental Figure S3. Glucose stimulated amino acid response in CTL and Fh1βKO islets
Supplemental Figure S4. Metabolite analysis with stable isotope tracer [U-$^{13}C_5$]-glutamine or $^{13}C_6$-glucose of murine Fh1βKO islets and CTL islets.
Supplemental Figure S5. Introduction of full length (FH) or cytoplasmic (FH^{cyt}) human fumarate hydratase restores mitochondrial morphology in β-cells lacking *Fh1*.
Supplemental Figure S6. Evidence of elevated protein succination in pancreatic islets from Fh1βKO mice and human diabetic donors.
Supplemental Figure S7. Glyceraldehyde blunts GSIS in CTL islets.
Supplemental Table 1. Metabolite analysis of murine Fh1βKO islets compared to CTL islets confirms *Fh1* loss leads to dysregulated metabolism.
Supplemental Table 2. Screening protein succination in Fh1βKO murine islets, non-diabetic and T2D human islets by mass spectrometry.

Supplemental Experimental Procedures

RNA isolation, reverse transcription and quantitative PCR (qPCR)

Total RNA was extracted from freshly-picked size-matched islets using Tri-reagent (Sigma) according to the manufacturer's protocol. Single-strand cDNA was synthesized from total RNA (2 µg) using a high-capacity cDNA kit (Applied Biosystems) and analyzed by multiplex qPCR using Taqman gene expression assays on a StepOne thermocycler (Applied Biosystems). Normalization was to β-actin mRNA and relative gene expression was calculated using the ΔΔCT method. DNA corresponding to 50 ng of RNA template was used and three biological replicates, each in duplicate, were performed for each experiment. Statistical comparisons were performed using an unpaired Student's *t* test.

Mouse Islet Isolation, culture and hormone secretion

Islets were isolated by collagenase (Sigma) or liberase (Roche) digestion. Collagenase or liberase was injected into the pancreas, which was then excised and incubated for 10-14 min at 37°C. Digestion was stopped by the addition of 10 ml of ice-cold Hank's Balanced Salt (HBSS; Sigma UK) supplemented with 0.1% bovine serum albumin (BSA) (Invitrogen, UK). The islets were left to sediment by gravity and washed further in HBSS with BSA to remove the remaining exocrine tissue. Mouse islets were transferred to RPMI-1640 (Sigma, UK) supplemented with 5 mM glucose, 100 U/ml penicillin, 10 µg/ml streptomycin and 10% fetal calf serum. Isolated mouse islets were cultured in this medium at 37 °C in a humidified atmosphere (5% CO_2/95% air) for 2 hr prior to experiments.

Hormone secretion was measured from batches of 10-12 islets and was counted as an experiment (unless otherwise stated). Size-matched islets were hand-picked and washed twice in glucose-free RPMI-1640 (Sigma; supplemented with 100 U/ml penicillin, 10 µg/ml streptomycin and 10% fetal calf serum). The islets were pre-incubated for 1 hr in a humidified chamber at 37 °C (5% CO_2/95% air) in 300µl of Krebs-Ringer buffer (KRB) which contained the following (mM) 140 NaCl, 3.6 KCl, 2.6 $CaCl_2$, 0.5 $MgSO_47H_2O$, 0.5 NaH_2PO_4, 2 $NaHCO_3$, 5 HEPES and 2 mg/ml BSA (pH adjusted to 7.4) and 1 mM glucose. The pre-incubation buffer was discarded and the islets were incubated for a further 1 hr, as indicated. In one set of experiments (Figure 3E), hormone secretion was measured in the presence of 70 mM KCl (indicated in the text) to bypass electrical activity and investigate the amplifying effect of glucose.

An aliquot of the supernatant was collected and stored at -20°C for quantification of insulin or glucagon secretion by radioimmunoassay. The remaining supernatant was discarded and the islets were lysed in 100 µl of ice-cold acid ethanol solution (containing ethanol, H_2O and HCl in a ratio of 52:17:1) to release their hormone content. The lysates were immediately frozen at -20 °C. Radio-immunoassays for insulin and glucagon were performed using kits (Millipore and Eurodiagnostica respectively) following the manufacturer's protocols.

Insulin and glucagon content of whole pancreas was determined by freezing and then grinding the pancreas, after weighing. This ground tissue was then transferred to ice-cold acid ethanol (as per above) and sonicated prior to determination of hormone content.

Whole-pancreas perfusion

Dynamic measurements of insulin secretion were performed using *in situ* pancreatic perfusion. Briefly, the aorta was cannulated by ligating above the coeliac artery and below the superior mesenteric artery, and the pancreas was perfused with KRB solution at a rate of ~0.45 ml/min using an Ismatec Reglo Digital MS2/12 peristaltic pump. The perfusate was maintained at 37 °C with a Warner Instruments temperature control unit TC-32 4B in conjunction with a tube heater (Warner Instruments P/N 64-0102) and a Harvard Apparatus heated rodent operating table. The effluent was collected, using a Teledyne ISCO Foxy R1 fraction collector. The pancreas was first perfused for 20 min with 1 mM glucose before commencing the experiment to establish the basal rate of secretion.

Membrane potential recordings

Freshly isolated mouse islets were immobilized by using a large bore glass electrode and electrical activity was measured from β cells on the periphery of the islets using an EPC-10 amplifier (HEKA Electronics) and Pulse software (version 8.81, HEKA Electronics). β-cells were identified by Na^+ current inactivation pattern and electrical activity in response to different glucose concentrations. In order to maintain metabolically intact cells, perforated patch whole-cell configuration was used. The extracellular solution is composed of (mM) 140 NaCl, 3.6 KCl, 0.5 $MgSO_4$, 0.5 NaH_2PO_4, 1.5 $CaCl_2$, 5 $NaHCO_3$ and 10 HEPES (pH7.4 with NaOH). The intracellular solution contained (mM) 76 K_2SO_4, 10 NaCl, 10 KCl, 1 $MgCl_2$ and 5 HEPES (pH 7.35 with KOH). Perforation

was achieved by the pore-forming antibiotic amphotericin B (at the final concentration of 40 µg/ml) added into the intracellular solution.

Measurement of [5-^{3}H]-glucose utilization and [U-^{14}C]-glucose oxidation
Groups of islets were incubated in KRB containing BSA (Sigma, 0.2%) and 1 or 20 mM glucose at 37 °C for 90 min in vials. Metabolism was stopped by the addition of HCl. The CO_2 produced during oxidation was captured in 400 µl phenylethylamine (Sigma), diluted 1:1 v/v in methanol, during a 90 min incubation period. The ^3H_2O produced was left to equilibrate to 2 ml water added to the vials, for a further 24 hr. Biodegradable scintillation liquid (10 ml) was then added to either the 400 µl phenylethylamine or to the 2 ml H_2O, in a scintillation vial. The $^{14}CO_2$ and the ^3H_2O were measured in a scintillation counter. Radiolabeled glucose and scintillation liquid were obtained from Perkin Elmer.

HPLC quantification of amino acids
Islet amino acids were separated by reverse-phase HPLC after pre-column derivatization with o-phthaldialdehyde and quantified by fluorescence detection. Groups of 30 islets were analyzed in triplicate from Stage II Fh1βKO and CTL littermates. The content and release of the measured islet amino acids were studied following previously published methods (Hernandez-Fisac et al, 2006). The residual incubation medium was aspirated, the islets were washed twice with 100 µl PBS and their amino acids extracted with 30 µl of 10% (w/v) 5-sulfosalicylic acid.

Metabolite analysis (CE-TOFMS) of islets
The levels and pattern of incorporation of the respective labels in metabolites (fmol/cell) were determined by capillary electrophoresis time of flight mass spectrometry (CE-TOFMS) analysis. Isolated Islets from individual mice of the same genotype were pooled and then separated into experimental groups. These islets were then washed and incubated at 37 °C in replicates under appropriate experimental conditions. Labelling studies were performed on islets isolated from Stage II Fh1βKO and age-matched CTL mice and cultured in RPMI-1640 (Sigma, UK) medium supplemented with 100 U/ml penicillin, 10 µg/ml streptomycin and 10% fetal calf serum containing either the stable isotope tracer [U-$^{13}C_5$]-glutamine for 3 hr or $^{13}C_6$-glucose for 1 hr at 1 or 20 mM glucose. At the end of the incubation, the islets were washed twice in an excess of 5% mannitol in water (Wako) and 200 µl of methanol was added containing 3 standards (methionine sulfone, 2-morpholinoethanesulfonate and D-Camphor-10-sulfonic acid, each at 25 µM). The islets were then left to rest for 10 min prior to freezing and storage at -80 °C. Frozen islet samples were prepared and analyzed by CE-TOFMS as described previously (Soga et al, 2006; Soga et al, 2009).

Electron microscopy and comparative quantitation of mitochondria
Isolated islets from Stage II CTL and Fh1βKO mice were fixed in 2.5% glutaraldehyde (Sigma, UK) in 0.1 M phosphate buffer, post-fixed in 1% osmium tetroxide, block stained in 2% uranyl acetate and embedded in Spurr's resin or London Resin Gold resin (LRG) (Agar Scientific, UK). Ultrathin sections were cut onto nickel grids, contrast was enhanced with 2% uranyl acetate and lead citrate and sections examined in a Joel 1010 microscope. For morphometry, mitochondrial cytoplasmic density and sectional area (size) were quantified from micrographs (x800 magnification) taken of 25-30 β cells in 3-5 islets from each experimental group.

Culture of human islets and CE-TOFMS
Human pancreata (Figure 5E and Table S2) were obtained with ethical approval and clinical consent from non-diabetic donors. Islets were isolated in the Diabetes Research & Wellness Foundation Human Islet Isolation unit (Oxford, UK) using modified versions of published procedures (Lake et al., 1989). Following isolation, islets were cultured in CMRL medium containing 5.5 mM glucose and 2 mM L-glutamine. Hand-picked islets were size-matched and cultured for 1 or 24 hr in RPMI containing glucose as indicated. Islets were pre-incubated in Krebs-Ringer buffer (KRB) containing 2 mg/ml BSA and 3 mM glucose for 1 hr at 37 °C, followed by a 1 hr test incubation in KRB supplemented with glucose as indicated. At the end of the incubation time the islets were processed for CE-TOFMS as described above for mouse islets.

Culture of human islets and GC/MS analysis
For these experiments (Figure 5F), human islets were obtained from the Nordic Center For Clinical Islet Transplantation (Uppsala, Sweden). Experimental procedures were approved by the Lund University Ethical Board. These were in compliance with the Declaration of Helsinki (2000) and the World Medical Association. Islets were processed according as previously described (Fadista et al., 2014). Islets were incubated 30 min at 2.8 mM glucose and then stimulated with either 2.8 or 16.7 mM glucose for 1 hr. The islets were transferred into 300 µl ice-cold extraction solvent. Metabolite extracts were analyzed on an Agilent 6890N gas chromatograph (Agilent Technologies, Atlanta, GA) equipped with an Agilent 7683B auto-sampler (Agilent Technologies) and

coupled to a LECO Pegasus III TOFMS electron impact time-of-flight mass spectrometer (LECO Corp., St. Joseph, MI).

Immunofluorescence and immunoblotting
Immunofluorescence was performed using the same antibodies as for IHC with Alexa Fluor® secondary antibodies (Molecular Probes, Life technologies) and an FITC-conjugated anti-V5 antibody (Life technologies) using a Zeiss LSM510 META confocal imaging system.

Immunoblotting was performed as described previously (O'Flaherty et al., 2010) using primary antibodies against FH (Autogen Bioclear) and calnexin (Calbiochem).

Mass spectrometry (MS) and Proteome analysis
Isolated pancreatic islets were lysed in 7 M Urea /SDS buffer and either run on an SDS-PAGE or processed in solution. For in solution samples, depletion of serum albumin was achieved by addition of ice-cold ethanol to a final concentration of 42% to the lysates and reconstitution of protein pellets in a buffer containing 6 M urea and 100 mM Tris (pH 7.8). Unmodified cysteine residues were alkylated in reducing conditions (10 mM DTT) using 30 mM 4-vinylpyridine (resulting in pyridylethylation, PE) or 50 mM iodoacetamide (carbamidomethylation, CA). Proteins were then subjected to trypsin digestion. In solution samples were further purified on Sep-Pak C18 columns (Waters).

Analysis of resulting peptides was performed on either an LTQ Orbitrap Velos or a Q-Exactive (Thermo Fisher Scientific).

LTQ Orbitrap Velos
Peptides were separated on an Acquity nano UPLC system (Waters) supplemented with a 25 cm C18 column, 1.7 µm particle size (Waters). They were eluted by applying a 60-180 min linear gradient from 1% buffer A (0.1% formic acid in water) to 40% buffer B (0.1% formic acid in acetonitrile) at a flow rate of 250 nl/min. Collision-induced dissociation (CID) was induced on the twenty most abundant ions per full MS scan using an isolation width of 1.5Da. All fragmented precursor ions were actively excluded from repeated MS/MS analysis for 15 s.

Q-Exactive
Peptides were separated on a Ultimate 3000 RSLCnano System utilizing a PepMap C18 column, 2 µm particle size, 75 µm x 50 cm (Thermo Scientific). A 60 min linear gradient was applied from 1% buffer A (0.1% formic acid, 5% DMSO in water) to 40% buffer B (0.1% formic acid, 5% DMSO in acetonitrile) at a flow rate of 250 nl/min. Collision-induced dissociation (CID) was induced on the fifteen most abundant ions per full MS scan using an isolation width of 1.5 Da. All fragmented precursor ions were actively excluded from repeated MS/MS analysis for 15 s.

All samples were analyzed in triplicate. Feature identification was performed by generation of Mascot generic files (mgf) using Proteowizard and analysis with either Mascot v2.3.01 or Peaks 7 searching the human or mouse SwissProt database.

Normalisation of MS runs and label-free quantitation of detected features was performed using Peaks 7 (Bioinformatics solutions). All features with a significant change between both conditions (ANOVA p-value < 0.05) were included in the analysis and imported to Ingenuity.

Supplemental References

Fadista, J., Vikman, P., Laakso, E.O., Mollet, I.G., Esguerra, J.L., Taneera, J., Storm, P., Osmark, P., Ladenvall, C., Prasad, R.B., et al. (2014). Global genomic and transcriptomic analysis of human pancreatic islets reveals novel genes influencing glucose metabolism. PNAS *111*, 13924-13929.

Hernandez-Fisac, I., Fernandez-Pascual, S., Ortsater, H., Pizarro-Delgado, J., Martin del Rio, R., Bergsten, P., Tamarit-Rodriguez, J. (2006). Oxo-4-methylpentanoic acid directs the metabolism of GABA into the Krebs cycle in rat pancreatic islets. Biochem J *400*, 81-89

Lake, S.P., Bassett, P.D., Larkins, A., Revell, J., Walczak, K., Chamberlain, J., Rumford, G.M., London, N.J., Veitch, P.S., Bell, P.R. et al. (1989). Large-scale purification of human islets utilizing discontinuous albumin gradient on IBM 2991 cell separator. Diabetes *38* Suppl 1, 143-145.

O'Flaherty, L., Adam, J., Heather, L.C., Zhdanov, A.V., Chung, Y.L., Miranda, M.X., Croft, J., Olpin, S., Clarke, K., Pugh, C.W. et al. (2010). Dysregulation of hypoxia pathways in fumarate hydratase-deficient cells is independent of defective mitochondrial metabolism. Hum Mol Genet *19*, 3844-3851.

Soga, T., Baran, R., Suematsu, M., Ueno, Y., Ikeda, S., Sakurakawa, T., Kakazu, Y., Ishikawa, T., Robert, M., Nishioka, T. et al. (2006). Differential metabolomics reveals ophthalmic acid as an oxidative stress biomarker indicating hepatic glutathione consumption. J Biol Chem *281*, 16768-16776.

Soga, T., Igarashi, K., Ito, C., Mizobuchi, K., Zimmermann, H.P., Tomita, M. (2009). Metabolomic profiling of anionic metabolites by capillary electrophoresis mass spectrometry. Anal Chem *81*, 6165-6174.

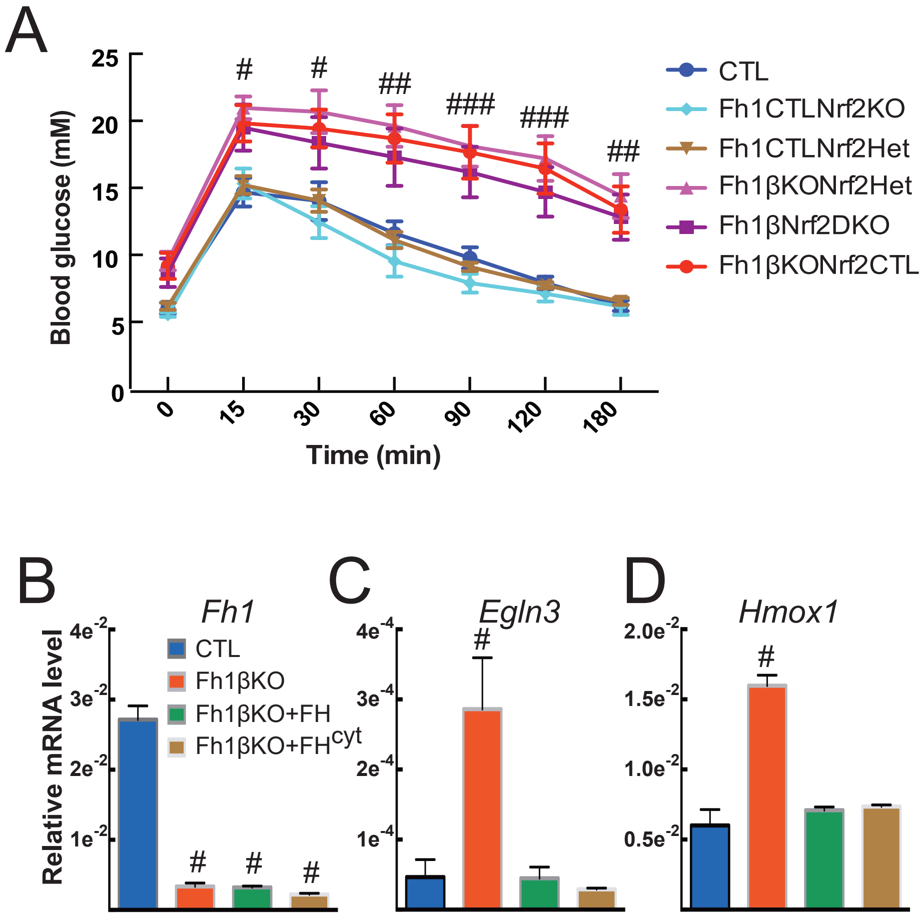

Figure S1. Introduction of full length (FH) and cytoplasmic (FHcyt) human fumarate hydratase rescues dysregulated metabolism in Fh1βKO islets. Related to Figure 1.

(**A**) Deletion of *Nrf2* did not alter the glucose tolerance in Stage II Fh1βKO compared to littermate CTL mice. IPGTT was performed in Fh1βKONrf2CTL, Fh1βKONrf2Het, Fh1βNrf2DKO and age matched CTL, Fh1CTLNrf2KO and Fh1CTLNrf2Het mice (n= 3 experiments using a total of at least 8 mice per genotype). #p<0.05, ##p<0.01 and ###p<0.001 between Fh1CTL and Fh1βKO groups irrespective of whether mice were heterozygous for *Nrf2* (Nrf2Het) or *Nrf2* was deleted (Nrf2KO).

(**B**-**D**) Analysis of gene expression by qPCR in isolated islets from Stage II CTL (blue), Fh1βKO (red), Fh1β KO+FH (green) and Fh1βKO+FHcyt (brown) mice for murine fumarate hydratase, *Fh1* (**B**), Egl-9 family hypoxia-inducible factor 3, *Egln3* (**C**), and heme oxygenase-1, *Hmox1* (**D**) (n=3 experiments using islets from at least 9 mice per genotype). These data confirm that although murine *Fh1* remains deleted in the β-cells of Fh1βKO, Fh1βKO+FH and Fh1βKO+FHcyt mice, the *Hif1α* target gene *Egln3* and the *Nrf2* target gene *Hmox1* both show mRNA expression in islets from Fh1βKO+FH and Fh1βKO+FHcyt mice comparable to that of CTL islets by virtue of expression of both full length and cytoplasmic-specific human FH. In contrast, the mRNA levels of *Egln3* and *Hmox1* are elevated in the Fh1βKO islets because of elevated fumarate leading to the subsequent stabilization of both HIF1α and NRF2. mRNA levels were normalized to those of Actb. #p<0.0001 versus CTL. Error bars indicate ± SEM.

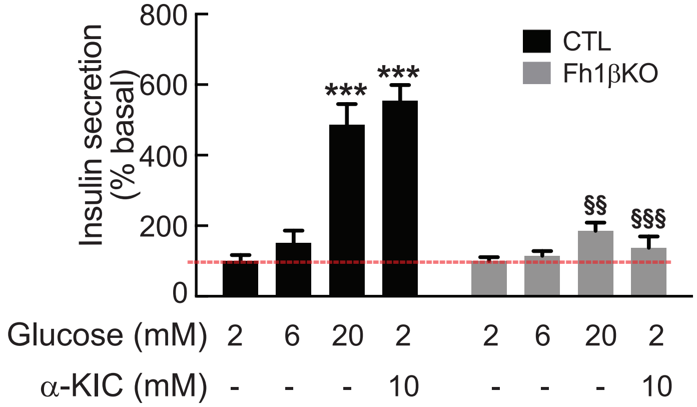

Figure S2. Glucose stimulated insulin secretion is blunted in Fh1βKO islets. Related to Figure 2.
Insulin secretion in age-matched CTL and Stage II Fh1βKO islets following 1 hr stimulation with glucose or α-ketoisocaproic acid (α-KIC; 10 mM). Results are displayed with insulin secretion expressed normalized to a percentage of the basal value (2 mM glucose). Approximate basal secretion is indicated (dashed line), (n=islets from at least 3 mice per genotype). ***$p<0.001$ versus 2 mM glucose CTL, §§$p<0.01$, §§§$p<0.001$ for comparison of the same condition between FH1βKO and CTL. Error bars indicate ± SEM.
Figures in the main text display insulin secretion data as absolute values.
Data have been normalized to the basal insulin release to compensate for any decrease in secretion due to lowered insulin content. The fact that stimulated insulin secretion remains strongly reduced in Fh1βKO islets argues that there is a functional defect.

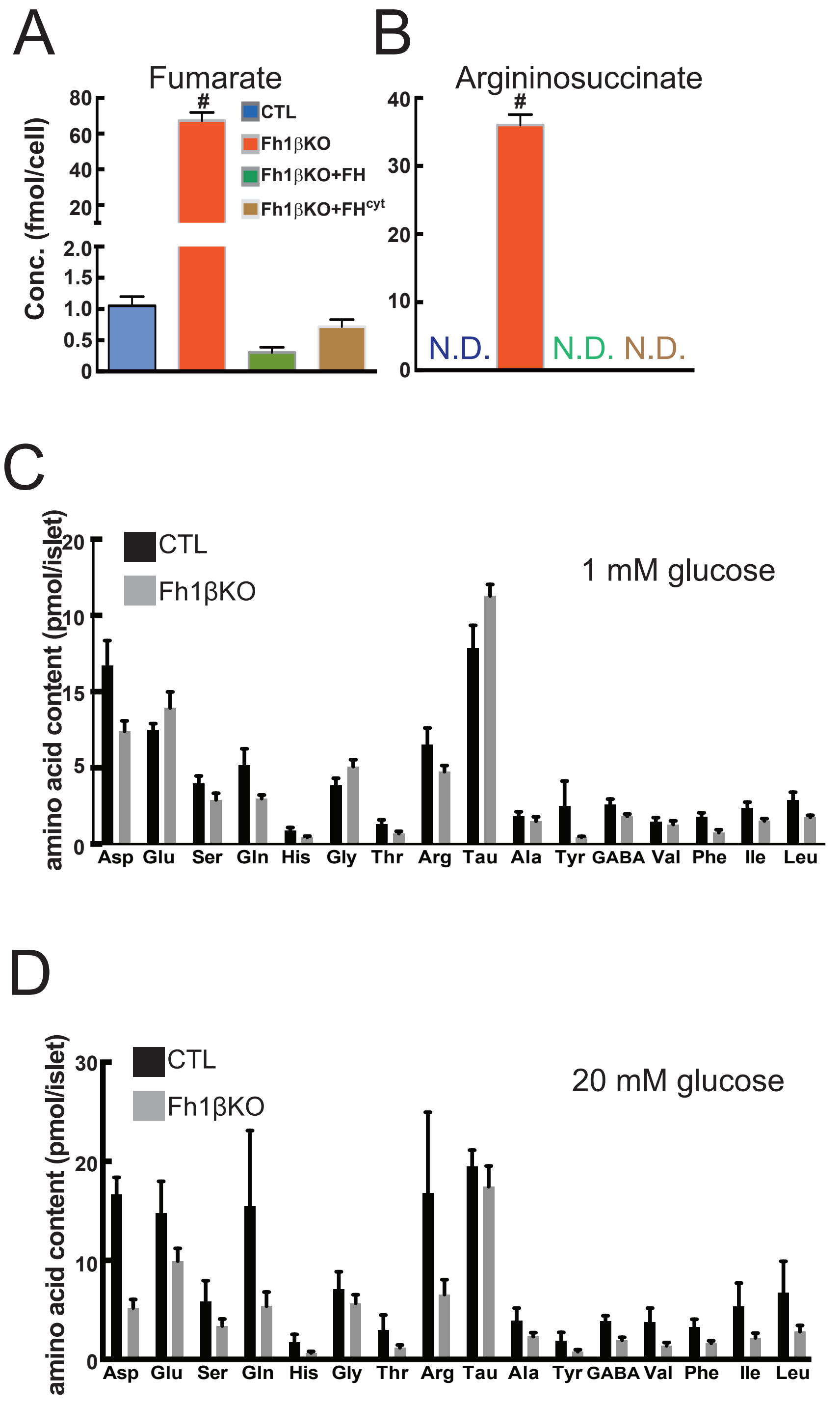

Figure S3. Glucose stimulated amino acid response in CTL and Fh1βKO islets. Related to Figure 3.
(**A-B**) Analysis by CE-TOFMS of islet content of significant metabolites from Stage II CTL (blue), Fh1βKO (red), Fh1βKO+FH (green) and Fh1βKO+FHcyt (brown) mice demonstrated that β cell fumarate (**A**) and argininosuccinate (**B**) contents were normalized to levels comparable to CTL mice following re-expression of full-length or cytoplasmic-specific human FH. #$p<0.0001$ versus CTL; ND not detected. (n=3 samples from 12-15 mice per experimental group).
(**C-D**) Amino acid content measured by HPLC in Stage II CTL (n=8 mice) and Fh1βKO (n=12 mice) islets after culture in 1 mM glucose (**C**) or 20 mM glucose (**D**) for 1 hr. (n=30 islets per genotype were analyzed in triplicate in 5 experiments). Abbreviations: Asp, aspartate; Glu, glutamic acid; Ser, serine; Gln, glutamine; His, histidine; Gly, glycine; Thr, threonine; Arg, arginine; Tau, taurine; Ala, alanine; Tyr, tyrosine; GABA, gamma aminobutyric acid; Val, valine; Phe, phenylalanine; Ile, isoleucine and Leu, leucine. Error bars represent ± SEM.

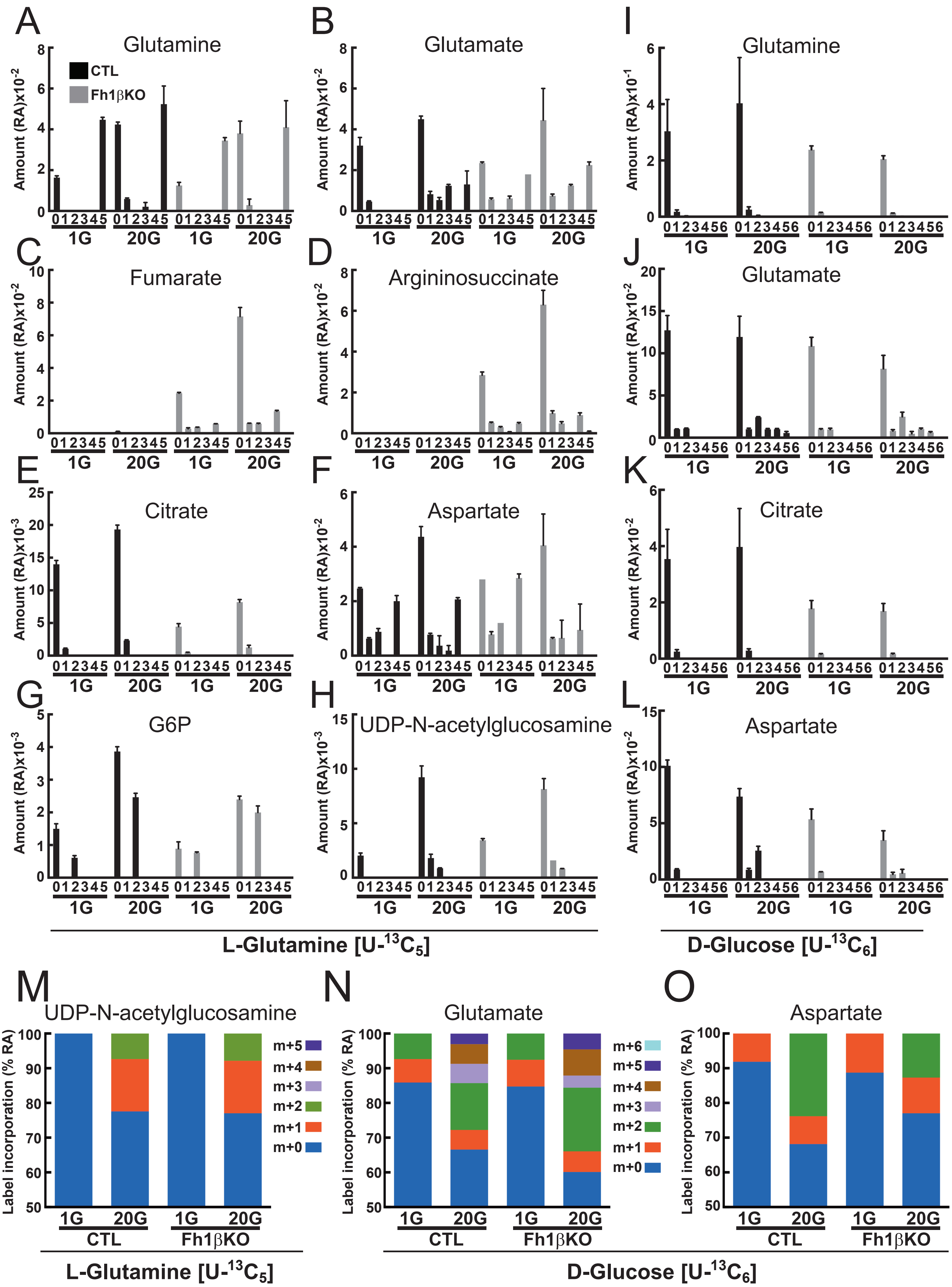

A
Glutamine
CTL
Fh1βKO
B
Glutamate
I
Glutamine
C
Fumarate
D
Argininosuccinate
J
Glutamate
E
Citrate
F
Aspartate
K
Citrate
G
G6P
H
UDP-N-acetylglucosamine
L
Aspartate
Amount (RA)x10^-2
Amount (RA)x10^-3
Amount (RA)x10^-1
1G
20G
L-Glutamine [U-13C5]
D-Glucose [U-13C6]
M
UDP-N-acetylglucosamine
N
Glutamate
O
Aspartate
Label incorporation (% RA)
m+0
m+1
m+2
m+3
m+4
m+5
m+6
CTL
Fh1βKO
L-Glutamine [U-13C5]
D-Glucose [U-13C6]

Figure S4. Metabolite analysis with stable isotope tracer [U-$^{13}C_5$]-glutamine or $^{13}C_6$-glucose of murine Fh1βKO islets and CTL islets. Related to Figure 3.
(**A-O**) Analysis by CE-TOFMS of metabolite levels (relative area) and the pattern of ^{13}C incorporation in islets isolated from age-matched CTL (black) and Stage II Fh1βKO (grey) mice following culture in medium containing either[U-$^{13}C_5$]-glutamine for 3 hr (**A-H** and **M**) or $^{13}C_6$-glucose for 1 hr (**I-L** and **N-O**) in cells and stimulation at 1 or 20 mM glucose. The samples are underivatized and uncorrected for natural abundance of ^{13}C.
(**M**) Changes in the percentage incorporation by glucose stimulation (1 and 20 mM glucose; 1G and 20G) of glutamine isotopomers (m0 to m+5) into UDP-N-acetylglucosamine following culture for 3 hr of isolated CTL and Fh1βKO islets in medium containing the stable isotope [U-$^{13}C_5$]-glutamine.
(**N**) Changes in the percentage incorporation by glucose stimulation (1 and 20 mM glucose) of glucose isotopomers (m0-m+6) into glutamate following culture for 1 hr of isolated CTL and Fh1βKO islets in medium containing the stable isotope $^{13}C_6$-glucose.
(**O**) Changes in the percentage incorporation by glucose stimulation (1 and 20 mM glucose) of glucose isotopomers (m0-m+6) into aspartate following culture for 1 hr of isolated CTL and Fh1βKO islets in medium containing the stable isotope ^{13}C-glucose.
(n=3 experimental groups of pooled islets for each experimental condition from at least 3 mice per genotype).

These data indicate that at 1 mM glucose none of the intracellular glutamate is derived from glutamine in CTL islets compared to ~50% in Fh1βKO islets. At 20 mM glucose, both the CTL and Fh1βKO islets use glutamine to produce glutamate. The small amount of islet tissue that could be isolated precluded a broad-spectrum analysis. However, at 1 mM glucose (1G) essentially none of the glutamate in the CTL islets came from labelled glutamine. By contrast, ~50% of the glutamate is derived from glutamine in Fh1βKO islets as label travels via the oxidative route of the Krebs cycle into fumarate and argininosuccinate. Most of this is from the direct conversion of glutamine to glutamate (m+5), but detection of m+3 may result from entry of fumarate into the urea cycle, allowing Asp m+4 to be produced. At 20 mM glucose, both the CTL and Fh1βKO islets use glutamine to produce glutamate, and both m+5 and m+3 are observed.

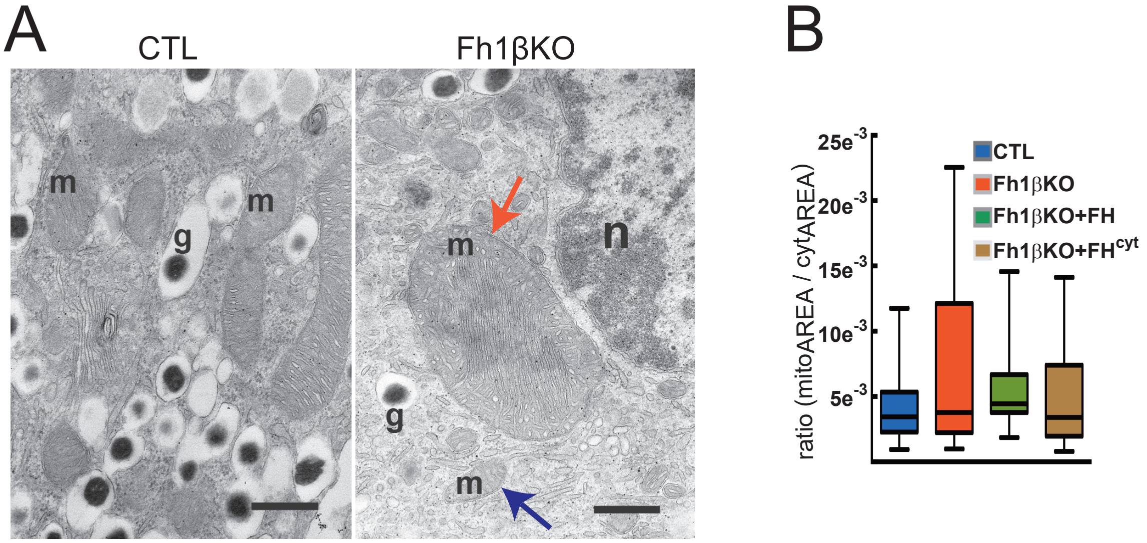

Figure S5. Introduction of full length (FH) or cytoplasmic (FHcyt) human fumarate hydratase restores mitochondrial morphology in β cells lacking *Fh1*. Related to Figure 4.

(**A**) Electron micrographs of β cells in Stage II CTL and Fh1βKO islets highlighting some very large mitochondria (red arrow) and smaller mitochondria (blue arrow) in the Fh1βKO β cells compared to CTL islets. Abbreviations: g, secretory granule; m, mitochondrion; n, nucleus. Scale bar: 500 nm.

(**B**) Relative mitochondrial area (mitochondrial area divided by cytoplasmic area; maximum to minimum range) compared in β cells in Stage II CTL (blue), Fh1βKO (red), Fh1βKO+FH (green) and Fh1βKO+FHcyt (brown) mice showing the larger range of mitochondrial areas in Fh1βKO islets and normalization following reintroduction of FH. (n=25-30 β cells in 3-5 islets per genotype). Error bars represent ± SEM.

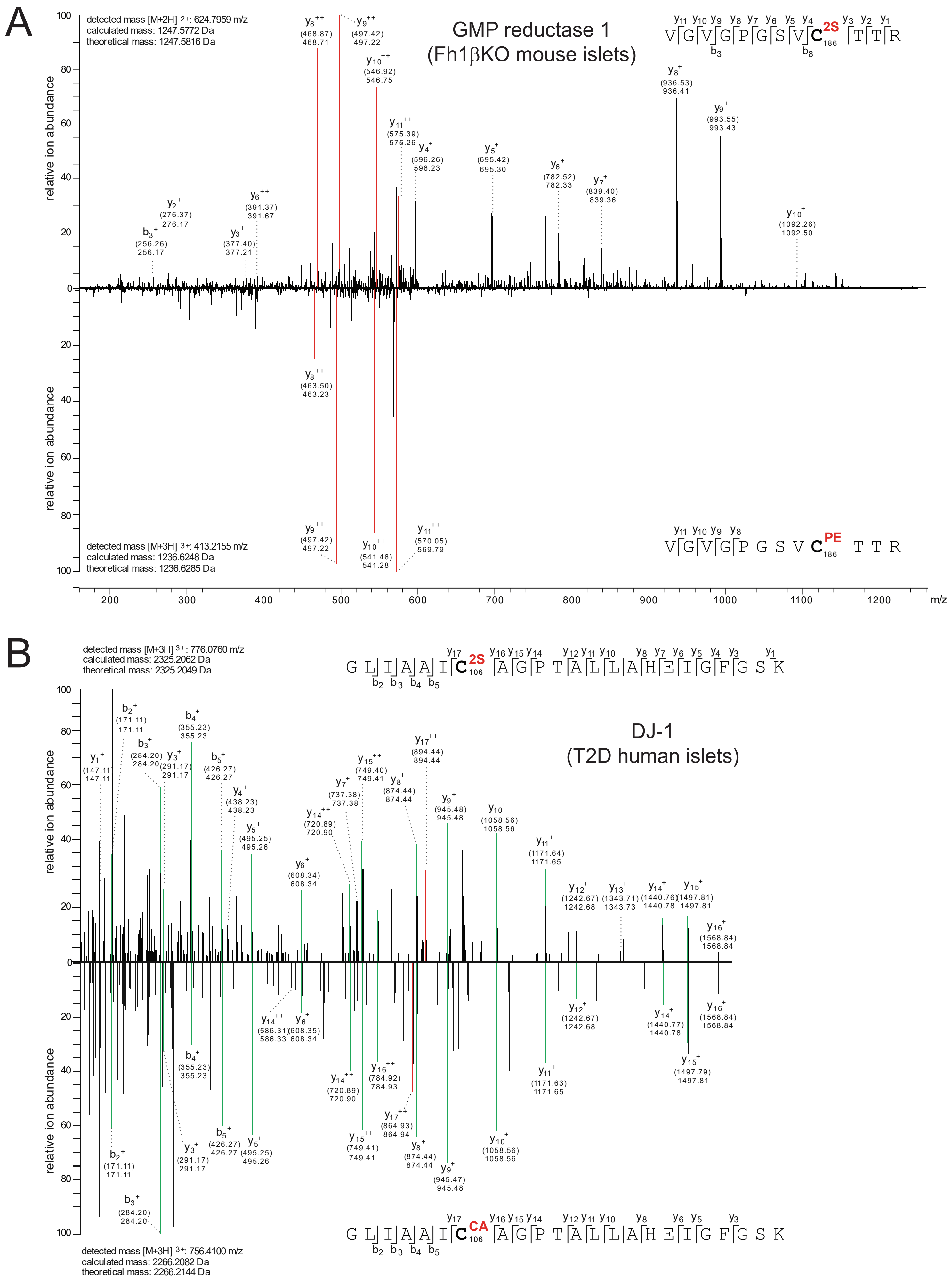

A
detected mass [M+2H] 2+: 624.7959 m/z
calculated mass: 1247.5772 Da
theoretical mass: 1247.5816 Da
GMP reductase 1
(Fh1βKO mouse islets)
VGVGPGSVC2S186TTR
detected mass [M+3H] 3+: 413.2155 m/z
calculated mass: 1236.6248 Da
theoretical mass: 1236.6285 Da
VGVGPGSVCPE186TTR
relative ion abundance
m/z
B
detected mass [M+3H] 3+: 776.0760 m/z
calculated mass: 2325.2062 Da
theoretical mass: 2325.2049 Da
GLIAAIC2S106AGPTALLAHEIGFGSK
DJ-1
(T2D human islets)
detected mass [M+3H] 3+: 756.4100 m/z
calculated mass: 2266.2082 Da
theoretical mass: 2266.2144 Da
GLIAAICCA106AGPTALLAHEIGFGSK
relative ion abundance
m/z

Figure S6. Evidence of elevated protein succination in pancreatic islets from Fh1βKO mice and human diabetic donors. Related to Figure 5.
(**A**) MS/MS spectra for peptide $_{178}$VGVGPGSVCTTR$_{189}$ showing increased succination in guanosine 5′-monophosphate oxidoreductase (GMP reductase 1) at cysteine 186 (C186) in Stage II Fh1βKO islets. Both theoretical mass and detected mass (in brackets) are given for each assigned fragment ion. Peptide fragments of different mass that contain the modified residue are highlighted in red. PE indicates the modification of cysteine residues at C186 to pyridylethyl-cysteine.
(**B**) MS/MS spectra showing either succination (^{2S}C) or carbamidoethylation (^{CA}C) at cysteine 106 (C106) in the $_{100}$GLIAAICAGPTALLAHEIGFGSK$_{122}$ peptide of human DJ-1 derived from islets of a T2D donor. Matching fragment ion peaks between the two peptide species that do not contain the modified residue are highlighted in green, whereas peptide fragments of different mass that contain the modified residue are highlighted in red.
The calculated peptide mass based on the detected m/z (m: mass, z: charge) value of the doubly charged precursor peptide ion ($[M+2H]^{++}$) and the calculated ([M] calc.) and theoretical peptide mass ([M] theor.) are stated for both peptide species. Detected N- and C-terminal fragment ions are indicated in the peptide sequence, assigned in the spectrum and depicted as follows: b: N-terminal fragment ion; y: C-terminal fragment ion; *: fragment ion minus NH_3; and $^{++}$: doubly charged fragment ion. Both theoretical mass (in brackets) and detected mass are given for each assigned fragment ion.

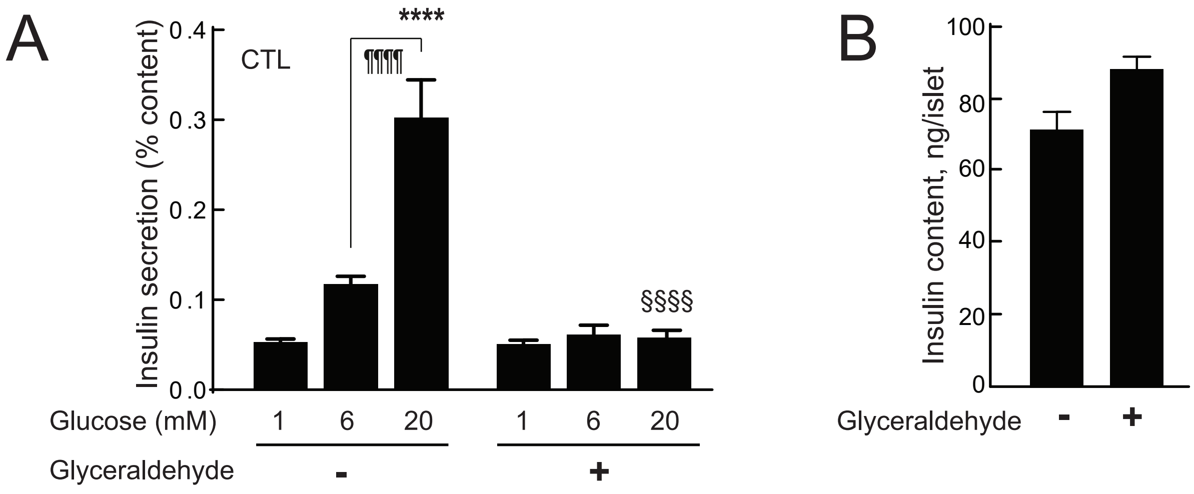

Figure S7. Glyceraldehyde blunts GSIS in CTL islets. Related to Figures 5 and 6.
(**A**) Insulin secretion measured during 1 hr incubations at 1, 6 and 20 mM glucose following 24 hr culture of wild-type islets from C57BL/6J mice with 10 mM glyceraldehyde. (n=6 experimental groups of islets for each condition assayed in 2 experiments; islets harvested from 6 mice in total). Data are mean values ± SEM of secretion expressed as per cent of hormone content. ****p<0.0001 versus 1 mM glucose; ¶¶¶¶p<0.0001 versus 6 mM glucose; §§§§p<0.0001 comparing same condition between groups.
(**B**) Insulin content of the islets in A.

Metabolite	WT	WT	WT	WT	WT	WT	WT	WT	KO	KO	KO	KO	KO	KO	KO	T-test
**Argininosuccinate**	0.00E+00	0.00E+00	0.00E+00	0.00E+00	0.00E+00	0.00E+00	0.00E+00	0.00E+00	9.19E-01	8.75E-01	9.39E-01	9.58E-01	9.13E-01	9.57E-01	8.28E-01	**8.72E-17**
**Fumarate**	6.53E-02	7.15E-02	1.22E-01	6.28E-02	6.81E-02	1.34E-01	7.82E-02	5.63E-02	1.20E+00	1.05E+00	1.06E+00	1.15E+00	1.21E+00	1.25E+00	1.16E+00	**1.45E-14**
**Glucose-6-phosphate**	2.46E-02	5.92E-02	4.63E-02	3.91E-02	3.26E-02	3.90E-02	3.86E-02	3.21E-02	0.00E+00	0.00E+00	0.00E+00	0.00E+00	0.00E+00	0.00E+00	3.39E-02	**7.42E-05**
**Aspartate**	1.26E+00	1.41E+00	1.54E+00	1.31E+00	1.19E+00	1.41E+00	1.33E+00	1.17E+00	1.06E+00	8.90E-01	8.96E-01	1.09E+00	1.09E+00	1.02E+00	1.08E+00	**1.09E-04**
**Adenylosuccinate**	0.00E+00	0.00E+00	0.00E+00	0.00E+00	0.00E+00	0.00E+00	0.00E+00	0.00E+00	7.07E-02	6.39E-02	5.89E-02	1.99E-01	7.08E-02	8.88E-02	1.44E-01	**1.27E-04**
**Cysteine-glutathione disulphide -Divalent**	8.49E-02	1.02E-01	1.09E-01	6.87E-02	3.75E-02	9.96E-02	6.01E-02	9.32E-02	6.97E-02	5.69E-02	0.00E+00	1.80E-02	4.40E-02	2.47E-02	1.84E-02	**2.07E-03**
**Adenosine monophosphate**	3.85E-01	5.45E-01	5.70E-01	2.40E-01	3.68E-01	6.06E-01	4.10E-01	2.74E-01	1.13E-01	1.80E-01	1.26E-01	3.26E-01	2.23E-01	9.76E-02	2.97E-01	**2.29E-03**
**Cystathionine**	2.02E-01	2.18E-01	2.79E-01	1.91E-01	1.85E-01	2.90E-01	1.83E-01	3.62E-01	3.95E-01	3.60E-01	3.85E-01	3.51E-01	3.18E-01	2.70E-01	3.19E-01	**3.37E-03**
**Uridine monophosphate**	9.10E-02	1.35E-01	1.31E-01	6.77E-02	1.15E-01	1.75E-01	1.24E-01	8.39E-02	0.00E+00	0.00E+00	0.00E+00	1.06E-01	7.35E-02	3.50E-02	1.05E-01	**6.30E-03**
**Guanidinoacetate**	5.13E-02	1.23E-01	1.22E-01	6.01E-02	5.36E-02	9.18E-02	1.02E-01	9.68E-02	5.64E-02	4.67E-02	0.00E+00	6.31E-02	5.47E-02	5.22E-02	5.48E-02	**9.36E-03**
**Gamma aminobutyricacid**	5.33E-01	7.11E-01	8.16E-01	7.00E-01	5.59E-01	7.92E-01	8.52E-01	6.80E-01	5.38E-01	4.27E-01	5.24E-01	5.85E-01	6.51E-01	5.04E-01	6.46E-01	**1.21E-02**
**Proline**	4.20E-01	4.94E-01	6.34E-01	5.39E-01	3.69E-01	5.79E-01	5.25E-01	5.01E-01	5.25E-01	3.40E-01	3.57E-01	3.85E-01	4.12E-01	3.78E-01	3.84E-01	**1.32E-02**

Metabolite	WT	WT	WT	WT	WT	WT	WT	WT	KO	KO	KO	KO	KO	KO	KO	T-test
**cis-Aconitate**	3.12E-01	2.00E-01	8.11E-02	1.50E-01	1.14E-01	1.16E-01	1.74E-01	2.73E-01	2.48E-01	4.36E-01	6.46E-01	2.32E-01	3.24E-01	2.47E-01	2.88E-01	**1.61E-02**
**Guanosine monophosphate**	1.63E-01	2.64E-01	2.72E-01	1.30E-01	1.47E-01	3.75E-01	1.99E-01	1.62E-01	0.00E+00	1.29E-01	0.00E+00	2.25E-01	1.01E-01	0.00E+00	1.77E-01	**1.78E-02**
**Glycine**	7.74E-01	8.17E-01	1.13E+00	9.82E-01	7.38E-01	9.04E-01	9.38E-01	7.81E-01	1.11E+00	9.61E-01	1.05E+00	9.93E-01	1.08E+00	9.74E-01	9.92E-01	**2.21E-02**
**Glucose 1-phosphate**	9.78E-02	1.16E-01	1.47E-01	1.05E-01	5.91E-02	9.12E-02	9.92E-02	9.52E-02	8.20E-02	7.47E-02	6.40E-02	4.43E-02	1.01E-01	9.05E-02	5.39E-02	**3.03E-02**
**Isocitrate**	6.27E-01	4.60E-01	1.57E-01	4.03E-01	2.00E-01	1.47E-01	3.55E-01	4.76E-01	4.75E-01	7.13E-01	1.09E+00	4.79E-01	5.49E-01	4.15E-01	5.01E-01	**3.35E-02**
**Glycero-phosphorylcholine**	4.44E-01	5.25E-01	6.75E-01	6.77E-01	3.99E-01	6.25E-01	7.73E-01	6.96E-01	5.94E-01	5.88E-01	7.59E-01	8.09E-01	7.48E-01	9.04E-01	9.57E-01	**3.70E-02**

Table S1. Metabolite analysis of murine Fh1βKO islets compared to CTL islets confirms *Fh1* loss leads to dysregulated metabolism. Related to Figure 3.
Analysis by CE-TOFMS of islet cellular concentrations (fmol/cell) for metabolites. Islets were isolated from individual Stage II Fh1βKO (n=7) and age-matched CTL (n=8) mice and incubated for 1 hr in 5 mM glucose then prepared for CE-TOFMS as described above. Metabolites listed show significance of $p<0.05$ or better between CTL and Fh1βKO islets (Student's *t*-test).

Table S2. Screening protein succination in Fh1βKO murine islets, non-diabetic and T2D human islets by mass spectrometry. Related to Figure 5.

This list of proteins was established using a false discovery rate of 1%. Protein sequence was confirmed on http://www.uniprot.org/. Quantitative analysis of protein changes was determined using Peaks (Bioinformatics solutions) and the proteins that were detected with significant change of abundance between both conditions are listed. P is the probability that the match between the MS/MS query and the peptide sequence is random. (n=150 islets isolated from 3 Stage II Fh1βKO mice; approximately 150 islets from each of 2 diabetic and 2 nondiabetic adult donors).
