# Supplementary material for: Fumarate Hydratase Deletion in Pancreatic β Cells Leads to Progressive Diabetes
Source: Cell Rep. 2017 Sep 26;20(13):3135–48. doi: 10.1016/j.celrep.2017.08.093 (PMC5637167; doi:10.1016/j.celrep.2017.08.093)
Supplement: Document S2. Article plus Supplemental Information [file mmc3.pdf]

Cell Reports

Article

Fumarate Hydratase Deletion in Pancreatic β Cells Leads to Progressive Diabetes

Graphical Abstract

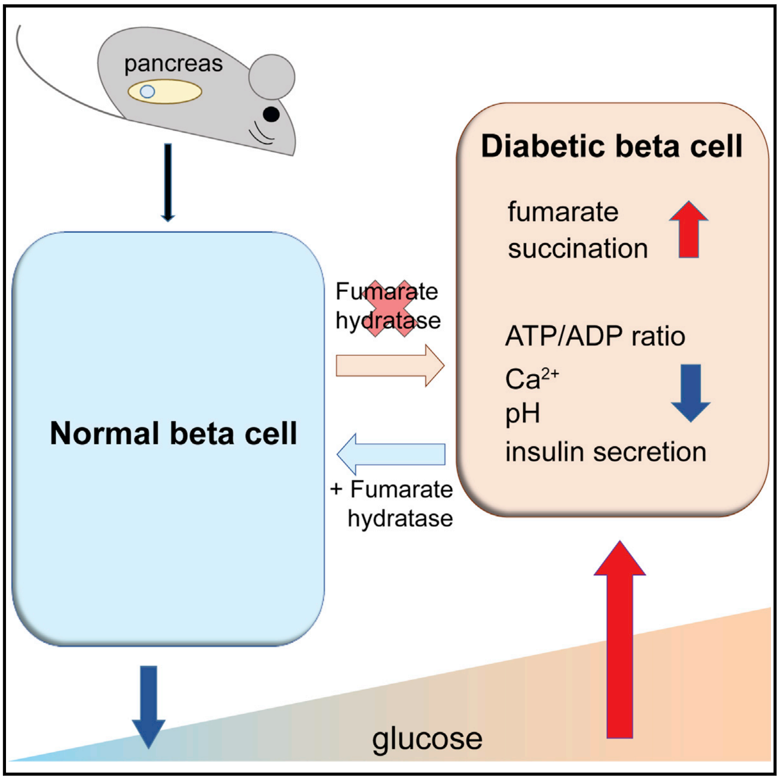

Authors

Julie Adam, Reshma Ramracheya, Margarita V. Chibalina, ..., Andrew Silver, Patrick J. Pollard, Patrik Rorsman

Correspondence

julie.adam@ndm.ox.ac.uk (J.A.), patrik.rorsman@hmc.ox.ac.uk (P.R.)

In Brief

Adam et al. have shown that progressive diabetes develops if fumarate hydratase is deleted in mouse pancreatic β cells. Such β cells exhibit elevated fumarate and protein succination and show progressively reduced ATP production and insulin secretion. The depleted insulin response to glucose recovers when diabetic islets are cultured in reduced glucose.

Highlights

- Fh1 loss in β cells causes progressive Hif1α-independent diabetes
- Fh1 loss in β cells impairs ATP generation, electrical activity, and GSIS
- Elevated fumarate is a feature of diabetic murine and human islets
- "Normoglycemia" restores GSIS in Fh1βKO islets

Adam et al., 2017, Cell Reports *20*, 3135–3148
September 26, 2017 © 2017 The Author(s).
http://dx.doi.org/10.1016/j.celrep.2017.08.093

Cell Reports
Article

OPEN ACCESS
CellPress

Fumarate Hydratase Deletion in Pancreatic β Cells Leads to Progressive Diabetes

Julie Adam,[1,2,3,16,18,*] Reshma Ramracheya,[1,16] Margarita V. Chibalina,[1,16] Nicola Ternette,[4] Alexander Hamilton,[1] Andrei I. Tarasov,[1] Quan Zhang,[1] Eduardo Rebelato,[1,5] Nils J.G. Rorsman,[1] Rafael Martín-del-Río,[6] Amy Lewis,[7] Gizem Özkan,[2] Hyun Woong Do,[1] Peter Spégel,[8] Kaori Saitoh,[9] Keiko Kato,[9] Kaori Igarashi,[9] Benedikt M. Kessler,[10] Christopher W. Pugh,[2,3] Jorge Tamarit-Rodriguez,[11] Hindrik Mulder,[12] Anne Clark,[1] Norma Frizzell,[13] Tomoyoshi Soga,[9] Frances M. Ashcroft,[14] Andrew Silver,[7] Patrick J. Pollard,[2,15] and Patrik Rorsman[1,15,17,*]
[1]Radcliffe Department of Medicine, OCDEM, Churchill Hospital, University of Oxford, Oxford OX3 7LE, UK
[2]Nuffield Department of Medicine, Henry Wellcome Building for Molecular Physiology, University of Oxford, Oxford OX3 7BN, UK
[3]Nuffield Department of Medicine, NDMRB, University of Oxford, Oxford OX3 7FZ, UK
[4]The Jenner Institute, Nuffield Department of Medicine, University of Oxford, Oxford OX3 7FZ, UK
[5]Department of Biophysics, Federal University of Sao Paulo, Sao Paulo 04023-062, Brazil
[6]Instituto Ramón y Cajal de Investigación Sanitaria (IRYCIS), Ramón y Cajal Hospital, Madrid, Spain
[7]Centre for Genomics and Child Health, Blizard Institute, Barts and The London School of Medicine and Dentistry, Queen Mary University of London, London E1 2AT, UK
[8]Centre for Analysis and Synthesis, Department of Chemistry, Lund University, Box 124, 221 00 Lund, Sweden
[9]Institute for Advanced Biosciences, Keio University, 246-2 Mizukami, Tsuruoka, Yamagata 997-0052, Japan
[10]Target Discovery Institute, Nuffield Department of Medicine, University of Oxford, Oxford OX3 7FZ, UK
[11]Biochemistry Department, School of Medicine, Complutense University of Madrid, 28040 Madrid, Spain
[12]Lund University Diabetes Centre, Unit of Molecular Metabolism, Clinical Research Centre, Malmo University Hospital, 20502 Malmo, Sweden
[13]Department of Pharmacology, Physiology & Neuroscience, School of Medicine, University of South Carolina, Columbia, SC 29208, USA
[14]Department of Physiology, Anatomy and Genetics, University of Oxford, Parks Road, Oxford OX1 3PT, UK
[15]Department of Physiology, Institute of Neuroscience and Physiology, University of Göteborg, 405 30 Göteborg, Sweden
[16]These authors contributed equally
[17]Senior author
[18]Lead Contact
*Correspondence: julie.adam@ndm.ox.ac.uk (J.A.), patrik.rorsman@hmc.ox.ac.uk (P.R.)
http://dx.doi.org/10.1016/j.celrep.2017.08.093

SUMMARY

We explored the role of the Krebs cycle enzyme fumarate hydratase (FH) in glucose-stimulated insulin secretion (GSIS). Mice lacking *Fh1* in pancreatic β cells (Fh1βKO mice) appear normal for 6–8 weeks but then develop progressive glucose intolerance and diabetes. Glucose tolerance is rescued by expression of mitochondrial or cytosolic FH but not by deletion of *Hif1α* or *Nrf2*. Progressive hyperglycemia in Fh1βKO mice led to dysregulated metabolism in β cells, a decrease in glucose-induced ATP production, electrical activity, cytoplasmic $[Ca^{2+}]_i$ elevation, and GSIS. Fh1 loss resulted in elevated intracellular fumarate, promoting succination of critical cysteines in GAPDH, GMPR, and PARK 7/DJ-1 and cytoplasmic acidification. Intracellular fumarate levels were increased in islets exposed to high glucose and in islets from human donors with type 2 diabetes (T2D). The impaired GSIS in islets from diabetic Fh1βKO mice was ameliorated after culture under normoglycemic conditions. These studies highlight the role of FH and dysregulated mitochondrial metabolism in T2D.

INTRODUCTION

Diabetes is an increasing and serious global health and financial problem (Ashcroft and Rorsman, 2012), characterized by defective insulin secretion from the β cells of the pancreatic islets, which causes elevated blood glucose. Mitochondrial production of ATP plays a key role in glucose-stimulated insulin secretion (GSIS) (Maechler and Wollheim, 1999); an increase in intracellular ATP closes ATP-sensitive K^+ channels (K_{ATP} channels) in the β cell plasma membrane, triggering depolarization and Ca^{2+}-dependent electrical activity. The resulting rise in cytoplasmic Ca^{2+} initiates exocytosis of insulin granules. In addition to this "triggering" effect, glucose amplifies insulin secretion at a stage subsequent to Ca^{2+} influx. Several intracellular factors might mediate this amplifying effect, including ATP, NADPH, and glutamate (Henquin, 2011).

The Krebs-cycle enzyme fumarate hydratase (FH) catalyzes the hydration of fumarate to malate. FH is also a tumor suppressor, mutated in hereditary leiomyomatosis and renal cell cancer (HLRCC) (Launonen et al., 2001). Loss of FH activity results in the intracellular accumulation of fumarate, the stabilization of hypoxia-inducible factor 1α (HIF1α), and activation of HIF-dependent pathways, including glucose metabolism (Adam et al., 2014).

The high levels of fumarate that accumulate in FH-deficient cells cause post-translational modification of cysteine residues in proteins to form S-(2-succino)-cysteine (2SC), a process

This is an open access article under the CC BY license (http://creativecommons.org/licenses/by/4.0/).

known as succination (Alderson et al., 2006). This induces loss of activity of the mitochondrial Krebs-cycle enzyme aconitase (Ternette et al., 2013), activation of the antioxidant response sensor nuclear factor (erythroid-derived 2)-like 2 (NRF2) (Adam et al., 2011), and elevation of reactive oxygen species (ROS) signaling (Sullivan et al., 2013; Zheng et al., 2015). Succination has been described in fat and skeletal muscle cells of some diabetic animal models (Nagai et al., 2007; Thomas et al., 2012). Its functional consequences include inactivation of the glycolytic enzyme glyceraldehyde 3-phosphate dehydrogenase (GAPDH) (Merkley et al., 2014; Blatnik et al., 2008).

HIF1α is a known regulator of GSIS (Girgis et al., 2012; Spégel et al., 2011). Deletion of von Hippel-Lindau protein (*Vhl*), an integral component of the HIF1α degradation pathway, in β cells leads to HIF1α stabilization, a switch from oxidative to glycolytic metabolism and consequent glucose intolerance (Zehetner et al., 2008; Cantley et al., 2009). The high levels of fumarate that result from FH loss competitively inhibit the 2-oxoglutarate-dependent dioxygenases that catalyze HIF prolyl hydroxylation. This allows HIF to escape degradation and results in the activation of HIF target genes (Isaacs et al., 2005; O'Flaherty et al., 2010), raising the possibility that FH loss might impair insulin secretion via HIF1α stabilization.

We explored the role of FH in insulin secretion using a mouse model in which *Fh1* was deleted specifically in pancreatic β cells (Fh1βKO mice). These mice had normal glucose tolerance for the first 6–8 weeks of life, and their β cells had essentially normal properties, including GSIS, despite the lack of a key Krebs-cycle enzyme. However, Fh1βKO mice subsequently developed rapidly progressing diabetes, culminating in severe glucose intolerance, reduced islet insulin content, and almost complete loss of GSIS.

RESULTS

Mice Lacking *Fh1* in Pancreatic β Cells Exhibit *Hif1α*-Independent Glucose Intolerance

We generated animals in which *Fh1* was deleted specifically in pancreatic β cells (*Fh1*$^{fl/fl}$*Rip2-Cre*$^{+/-}$; Fh1βKO mice) by intercrossing an *Fh1* conditional knockout mouse (Pollard et al., 2007) with mice expressing Cre recombinase driven by the rat insulin promoter (*Rip2-Cre* mice; Herrera, 2000). Control (CTL) mice were either *Fh1*$^{fl/fl}$*Rip2-Cre*$^{-/-}$ or *Fh1*$^{fl/+}$*Rip2-Cre*$^{+/-}$ littermates. Deletion of *Fh1* in β cells was confirmed at the protein level in islets of 9- to 12-week-old mice (Figure 1). No marked differences were seen in gross histology between CTL and Fh1βKO mice in islets (Figures 1A and 1F). Immunohistochemistry (IHC) showed that all cells in CTL islets exhibited uniform expression of FH, which was lost within the core of Fh1βKO islets. However, some islet cells, most likely α and β cells, retained FH (Figures 1B, 1G, and 1J). *Fh1* loss and elevated fumarate lead to stabilization of HIF1α and subsequent nuclear localization (Isaacs et al., 2005; Pollard et al., 2005). Figures 1C and 1H show nuclear localization of HIF1α in most cells of Fh1βKO islets, but not in CTL islets or the exocrine pancreas. No marked differences were observed in insulin or glucagon IHC between CTL and Fh1βKO islets (Figures 1D, 1E, 1I, and 1J).

Deletion of *Fh1* was confirmed in Fh1βKO islets from 9- to 12-week-old mice at the mRNA level. A small amount of *Fh1* mRNA remained in Fh1βKO islets, likely reflecting its presence in non-β cells (Figure 1K). Expression of the *Hif1α* target genes pyruvate dehydrogenase kinase 1 (*Pdk1*) and prolyl hydroxylase dehydrogenase 3 (*Phd3/Egln3*) was increased in Fh1βKO islets (Figure 1K).

Blood glucose levels were measured in free-fed CTL and Fh1βKO littermates from 6 to 20 weeks. While young (6–8 weeks of age) Fh1βKO mice were nearly normoglycemic, they subsequently (at >9 weeks of age) developed severe hyperglycemia (>20 mM) (Figure 1L). Plasma glucose in CTL mice was stable at all ages (<10 mM). For simplicity, here we refer to CTL and Fh1βKO littermates at 6–8, 9–12, and >15 weeks of age as stages I–III, respectively, to match the progressive diabetes in Fh1βKO mice. Stages I (non-diabetic [ND]), II (diabetic), and III (severely diabetic) have free-fed blood glucose levels of 12.8 mM ± 0.6 mM, 22.6 mM ± 1.2 mM, and 30.4 mM ± 0.9 mM (Figure 1L). The progression of diabetes was confirmed in glucose tolerance tests, which revealed mild intolerance between 9 and 12 weeks of age (stage II) and severe intolerance by 15 weeks (stage III) (Figures 1M and 1N). No age-dependent deterioration of glucose tolerance was observed in CTL mice.

To assess whether the glucose intolerance of Fh1βKO mice is mediated by HIF1α stabilization, we crossed Fh1βKO and *Hif1α*$^{fl/fl}$ mice (Cramer et al., 2003) to produce β cell-specific deletion of both *Fh1* and *Hif1α* (Fh1Hif1αβKO mice). Deletion of *Hif1α*, confirmed by loss of HIF1α staining in all nuclei of Fh1Hif1αβKO islets (Figure 1O), did not ameliorate the glucose intolerance of Fh1βKO mice (Figure 1P). Loss of *Fh1* also leads to stabilization of NRF2 and activation of downstream pathways, typified by increased expression of *Hmox1* (confirmed in Figure S1D) (Adam et al., 2011). The role of *Nrf2* in β cell function is unclear, proposed to protect from oxidative damage and blunt GSIS (Uruno et al., 2013). To determine the contribution of *Nrf2* to glucose homeostasis, we crossed Fh1βKO with a constitutive knockout of *Nrf2* (Itoh et al., 1997) to delete both *Fh1* and *Nrf2* in the β cell (Fh1βNrf2DKO). The glucose intolerance of Fh1βKO mice was unaltered in the Fh1βNrf2DKO mice (Figure S1A). In contrast, crossing Fh1βKO mice with mice stably expressing either full-length human FH (Fh1βKO+FH) or cytoplasmic FH (FH^{cyt}) (Fh1βKO+FH^{cyt}) (Adam et al., 2013) fully reversed the glucose intolerance tested in stage III mice and restored the mRNA expression of *Egln3* and *Hmox1* (Figures 1Q and S1B–S1D), and these mice were normoglycemic for >1 year.

Deletion of *Fh1* in β Cells Results in Progressive Loss of GSIS

To explore the cause of glucose intolerance in Fh1βKO mice, we examined insulin secretion from the perfused pancreas. Glucose elevation from 1 mM to 6 mM transiently stimulated insulin release ~10-fold in stage II CTL mice but not in Fh1βKO littermates (Figure 2A). Nevertheless, the response to 20 mM glucose was almost superimposable in both genotypes. In stage III Fh1βKO mice, the response to both 6 mM and 20 mM glucose was 85% less than in CTL (Figure 2B). Similar effects on GSIS were obtained in static incubations (Figure S2). The mitochondrial substrate α-ketoisocaproic acid (α-KIC) stimulated insulin secretion ~5-fold in CTL islets but had no effect in Fh1βKO islets (Figure S2).

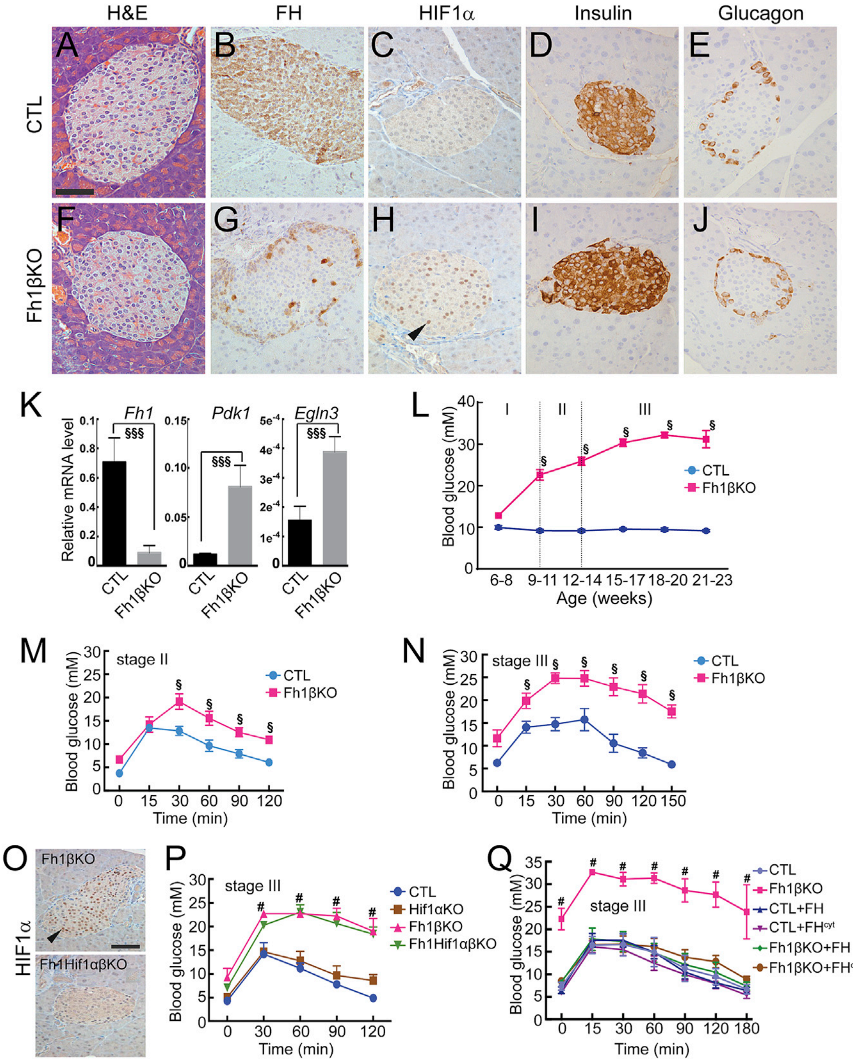

Figure 1. *Fh1* Loss in Pancreatic β Cells Results in Progressive *Hif1α*-Independent Glucose Intolerance

(A–J) Histological analysis of pancreatic islets from stage II CTL (A–E) and Fh1βKO (F–J) mice (n = at least 10 islets from each of 10 mice per genotype).

(A and F) H&E staining of CTL and Fh1βKO, respectively. (B–E and G–J) Immunohistochemistry (IHC) for FH (B and G), HIF1α (C and H), insulin (D and I), and glucagon (E and J) in CTL (B–E) and Fh1βKO (G–J). Scale bar, 50 μm (all panels).

(K) mRNA levels of *Fh1*, *Pdk1*, and *Egln3* relative to *Actb* (β-actin) in stage II CTL (black) and Fh1βKO islets (gray). (n = 30–50 islets in 5 experiments from a total of 10 animals of each genotype). $^{§§§}p < 0.0001$.

(L) Free-fed blood glucose measured in different aged CTL (blue) and Fh1βKO mice (red). Stages I, II, and III are identified (n > 200 Fh1βKO mice, and n > 50 CTL mice). $^{§}p < 0.0001$.

(M and N) Intraperitoneal glucose tolerance test (IPGTT) performed in (M) stage II CTL (blue; n = 15) and Fh1βKO (red; n = 10) mice and (N) stage III mice (n = 7 per group). $^{§}p < 0.05$.

(O) IHC for HIF1α in pancreas of stage III Fh1βKO (top) and Fh1HifαβKO (bottom) mice (n = at least 10 islets from each of 4 mice per genotype). Scale bar, 50 μm.

(P) IPGTT performed in stage III CTL, Fh1βKO, Hif1αKO, and Fh1Hif1αβKO mice. $^{\#}p < 0.05$, comparing Fh1βKO or Fh1Hif1αβKO versus CTL or Hif1αKO; Fh1βKO versus Fh1Hif1αβKO and CTL versus Hif1αKO are not significant (n = at least 5 mice per genotype).

(Q) Reintroduction of FH or FH^{cyt} rescued the glucose intolerance of Fh1βKO mice. IPGTT performed on stage III CTL, Fh1βKO, CTL+FH, CTL+FH^{cyt}, Fh1βKO+FH, and Fh1βKO+FH^{cyt} mice. $^{\#}p < 0.0001$, Fh1βKO versus all other groups (n = 6–8 mice per genotype).

Arrows in (H) and (O) point to nuclei. Error bars represent ± SEM. See also Figure S1.

These differences in insulin release correlated with a reduction in insulin content. In islets from stage II mice, insulin content and insulin granule density were reduced by ~50% in Fh1βKO islets (Figures 2C–2E). Pancreatic insulin content was 97% less in stage III Fh1βKO mice, compared to CTL littermates (Figure 2F), and there was a marked decrease in insulin-positive cells (Figure 2G). When insulin secretion data are normalized to basal values, secretion from stage II Fh1βKO islets remains strongly reduced, suggesting that there is a functional defect (Figure S2).

Fh1βKO Islets Exhibit Dysregulated Metabolism

As mitochondrial metabolism plays a key role in GSIS (Ashcroft and Rorsman, 2012) and *Fh1* deletion disrupts the Krebs cycle, we analyzed glucose utilization (^3H_2O production, reflecting combined flux through the glycolytic and pentose phosphate pathways) and oxidation (CO_2 production, reflecting mitochondrial metabolism) (Hellman et al., 1971). In both CTL and Fh1βKO stage II islets, glucose oxidation increased 4-fold when glucose was increased from 1 to 20 mM (Figure 3A). Glucose utilization also increased 4-fold, but the effect was 35%–40% greater in Fh1βKO than CTL islets (Figure 3B), suggestive of increased aerobic glycolysis (O'Flaherty et al., 2010).

Metabolite analysis in islets from stage II mice confirmed an overall pattern of changes similar to that observed in other FH-deficient cells (Adam et al., 2011; Frezza et al., 2011). In particular, levels of fumarate, argininosuccinate, and adenylosuccinate were increased, and aspartate was reduced (Figure 3C; Table S1), indicating that FH loss leads to stimulation of the urea cycle/arginine biosynthesis pathway (Adam et al., 2013; Zheng et al., 2013). Levels of AMP, uridine monophosphate (UMP), and guanosine monophosphate (GMP) were decreased, suggesting both purine and pyrimidine metabolism are compromised as a consequence of aspartate depletion, but likely reflect altered adenylosuccinate metabolism due to elevated fumarate (Bulusu et al., 2011). Importantly, no difference was observed in oxidized or reduced glutathione between CTL and Fh1βKO islets, suggesting no major change in ROS or ROS signaling (Sullivan et al., 2013). Islet fumarate and argininosuccinate content

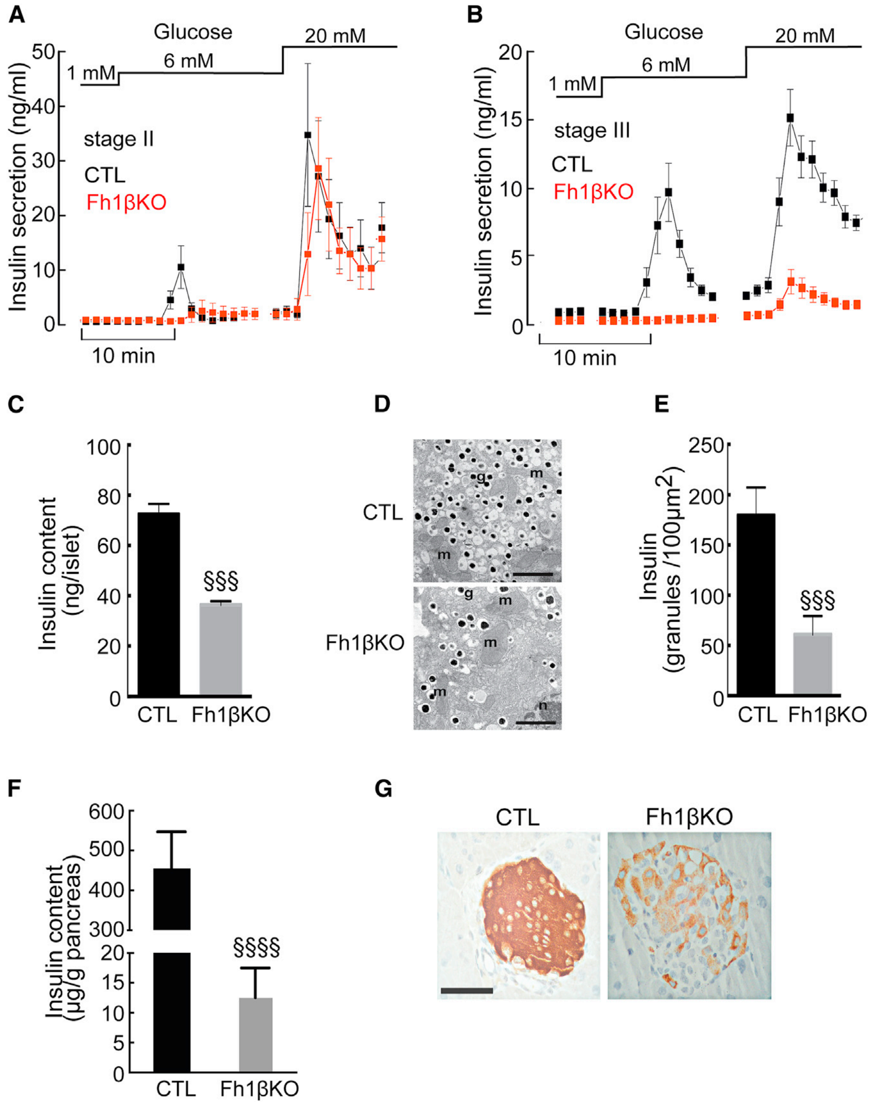

Figure 2. Deletion of *Fh1* in β Cells Impairs Insulin Secretion

(A and B) Insulin secretion from the perfused pancreata of stage II (A) and stage III (B) Fh1βKO mice (red) and CTL mice (black) (n = 3 mice for each genotype and age) in response to 1, 6, and 20 mM glucose. Statistical significances are omitted for clarity.

(C) Insulin content of islets from stage II CTL mice (black; n = 10 experiments; n = 10 mice) and Fh1βKO mice (gray; n = 14 experiments; n = 22 mice). §§§$p < 0.0001$.

(D) Electron micrographs of β cells from CTL and Fh1βKO mice. Abbreviations: g, insulin secretory granules; m, mitochondrion; n, nucleus. Scale bars, 500 nm.

(E) Insulin granule density measured in electron micrographs of β cells from stage II Fh1βKO (gray) and CTL (black) mice (25–30 β cells in 3–5 islets per genotype), §§§$p < 0.001$.

(F) Pancreatic insulin content of stage III CTL (black, n = 5) and Fh1βKO (gray, n = 10) mice. §§§§$p < 0.0001$.

(G) Representative examples of insulin IHC in stage III Fh1βKO and CTL islets (n > 100 islets from n > 10 mice of each genotype). Scale bar, 50 μm. Error bars represent ± SEM. See also Figure S2.

were normalized following re-expression of either FH or FH^{cyt} in mice (Figures S3A and S3B).

The lack of FH means there is a loss of Krebs-cycle intermediates for every glucose molecule entering the mitochondria. Since metabolism might be maintained by enhanced utilization of amino acids (anaplerosis), we measured 16 key amino acids in islets at both 1 mM and 20 mM glucose. Glucose elevation increased levels of 15 amino acids in CTL islets but had little effect in stage II Fh1βKO islets (Figures 3D, S3C, and S3D).

Glutamate may play a key role in both glucose- and incretin-induced insulin secretion, via the amplifying (non-K_{ATP}-dependent) pathway (Maechler and Wollheim, 1999). Because the glucose-induced increase in glutamate was lower in Fh1βKO islets than in CTL islets (Figure 3D), we investigated the amplifying pathway of insulin secretion using islets from stage II animals depolarized with 70 mM extracellular K^+ (to test the amplifying effect of glucose). Increasing glucose from 2 mM to 20 mM amplified insulin secretion by 600% in CTL islets but only by 150% in Fh1βKO islets (Figure 3E). Addition of exogenous membrane-permeable dimethyl glutamate (DMG; 5 mM) had no effect in CTL islets at either 2 mM or 20 mM glucose but potentiated GSIS in Fh1βKO islets in response to 20 mM glucose (Figure 3E). This suggests that the lack of intermediates, including glutamate, may underlie the impaired insulin release of Fh1βKO islets. Exogenous membrane-permeable dimethyl fumarate (DMF; 5 mM) did not inhibit insulin secretion in CTL islets (Figure 3F), indicating that the acute increase of intracellular fumarate in Fh1βKO islets is not the cause of the impaired GSIS.

Some aspects of altered metabolism in Fh1βKO islets were also investigated by comparing glutamine (m0 to m+5) and glucose (m0 to m+6) isotopomers in islets from stage II Fh1βKO and CTL mice incubated in 1 mM or 20 mM glucose with either [U-$^{13}C_5$]-glutamine or $^{13}C_6$-glucose (Figure S4). Increased levels of fumarate and argininosuccinate, derived from glutamine, were observed in islets subsequently exposed to 20 mM glucose (Figures S4C and S4D). Islets cultured at 20 mM glucose utilized glutamine to generate uridine 5'-diphosphate (UDP)-N-acetylglucosamine (Figures S4H and S4M), a precursor for synthesis of glycosaminoglycans, proteoglycans, and glycolipids (Bond and Hanover, 2013). Substitution of the isotopomers m+2, m+3, m+4, and m+5 for the naturally occurring m+0 and m+1 forms was increased significantly in both glutamate and aspartate in both CTL and Fh1βKO in islets incubated with 20 mM glucose (Figures S4J, S4L, S4N, and S4O).

OPEN
ACCESS
CellPress

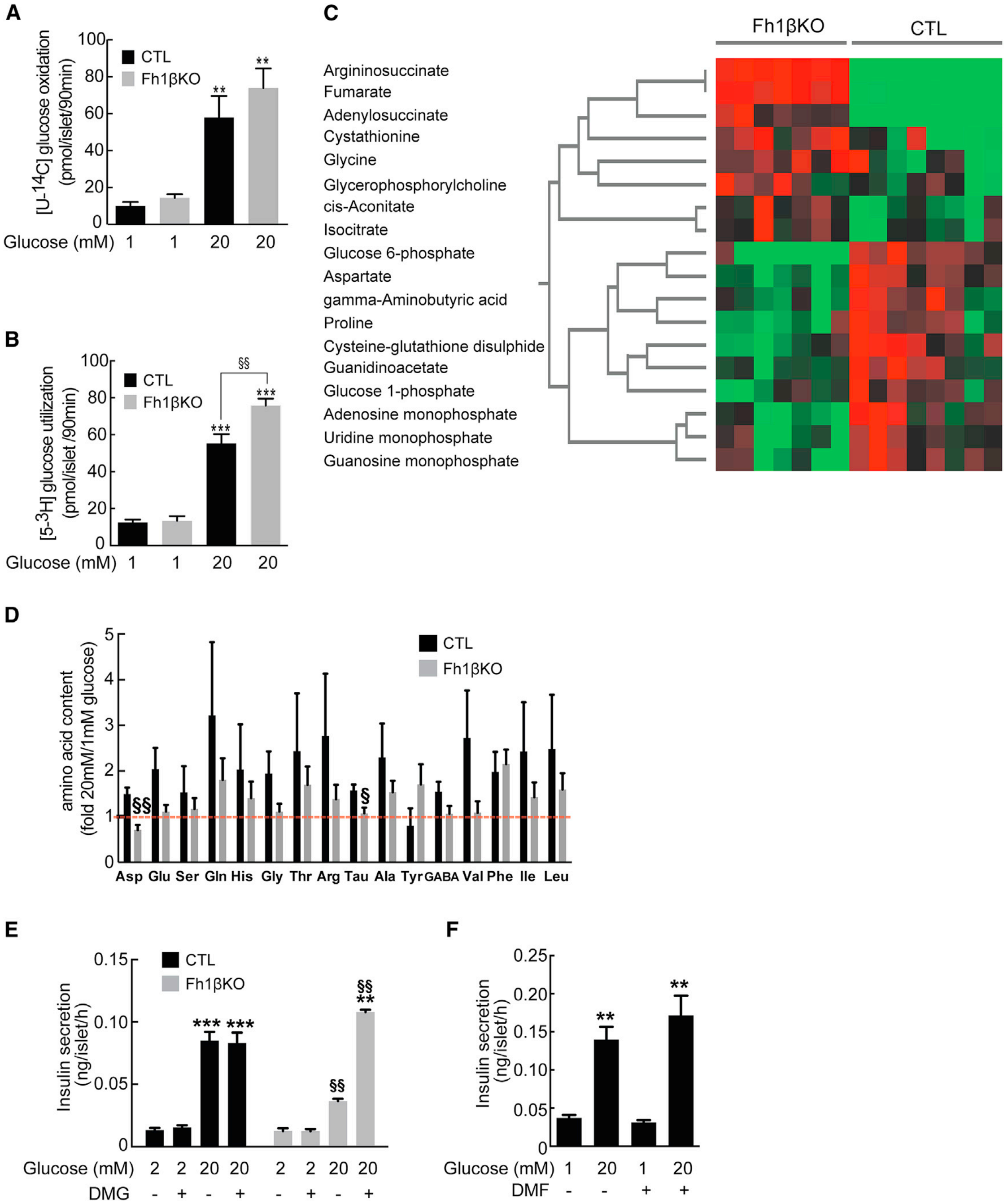

Figure 3. Ablation of *Fh1* Causes Dysregulated Metabolism

(A) Glucose oxidation in stage II Fh1βKO and CTL islets (n = 7 CTL and n = 16 Fh1βKO mice in 3 experiments) at 1 mM and 20 mM glucose. $^{**}p < 0.01$ versus 1 mM glucose; not significant between CTL and Fh1βKO islets.

(B) Glucose utilization in CTL and Fh1βKO islets from stage II mice (n = 7 mice of each genotype in 3 experiments) at 1 mM and 20 mM glucose. $^{***}p < 0.001$ versus 1 mM glucose; $^{§§}p < 0.01$ between CTL and Fh1βKO at 20 mM glucose.

(C) Heatmap of metabolites (measured by CE-TOFMS) that show significant concentration differences between islets from stage II CTL (n = 8 mice) and Fh1βKO (n = 7 mice) mice incubated at 5 mM glucose for 1 hr. Red and green for Fh1βKO islets indicate metabolites that are significantly increased or decreased, respectively, versus CTL islets ($p < 0.05$); Student's t test. Each column represents values for islets from one animal. Absolute values are given in Table S1. Branch points indicate metabolites linked in common pathways.

(D) Amino acid content in islets of stage II Fh1βKO (gray; n = 12) and CTL (black; n = 8) mice at 20 mM glucose, expressed relative to that at 1 mM glucose (20G/1G) (n = 30 islets per group analyzed in triplicate in 5 experiments). Abbreviations: Asp, aspartate; Glu, glutamic acid; Ser, serine;Gln, glutamine; His, histidine; Gly,

(legend continued on next page)

Progressive Loss of GSIS in Fh1βKO Mice Correlates with Reduced ATP Production and β Cell Electrical Activity

Next, we compared the effects of glucose on ATP production and electrical activity in CTL and Fh1βKO mice. The glucose-induced increase in the ATP/ADP ratio was identical in stage I Fh1βKO and CTL islets (Figure 4A). However, β cells in islets from stage III Fh1βKO mice exhibited far smaller glucose-induced increases in the ATP/ADP ratio than age-matched CTL β cells (Figures 4B and 4C).

Glucose-induced electrical activity in β cells from stage II Fh1βKO mice was similar to that of CTL islets (Figure 4D). However, clear differences were observed in stage III mice. Whereas CTL β cells responded to 20 mM glucose with membrane depolarization and action potential firing, β cells in stage III Fh1βKO islets were refractory to glucose stimulation (Figure 4E). In such glucose-unresponsive Fh1βKO β cells, electrical activity was elicited by the K_{ATP}-channel blocker tolbutamide. Together, these data suggest that glucose is unable to inhibit K_{ATP} channels in stage III Fh1-deficient β cells, because ATP generation is reduced.

Fh1 Deletion Causes Disruption of β Cell Mitochondrial Ultrastructure

The morphology, size, and distribution of mitochondria in β cells of stage II mice were analyzed by electron microscopy. Whereas mitochondria in CTL β cells had normal morphology with clear cristae, many mitochondria in Fh1βKO β cells were swollen without clear cristae, and some were very large (>1.25 μm in diameter). There was larger range in the mitochondrial area in Fh1βKO β cells, suggesting an imbalance between mitochondrial fission and fusion (Figures S5A and S5B). Mitochondria in islets from Fh1βKO+FH (full-length FH rescue) or Fh1βKO+FH^{cyt} (cytoplasmic FH rescue) mice were similar to those of CTL mice (Figure S5B).

Protein Succination in Diabetic Islets

Fumarate reacts with cysteine residues in proteins in a non-enzymatic process known as succination. Using an antibody (2SC) that labels succinated proteins specifically (Blatnik et al., 2008), we confirmed that succination was detected in Fh1βKO β cells and not in CTL β cells (Figures 5A and 5B).

Culture of adipocytes in high glucose elevates fumarate and results in protein succination (Nagai et al., 2007). Therefore, we determined quantitatively whether hyperglycemia would increase fumarate in islets. Fumarate levels were ~100-fold higher in stage II Fh1βKO islets, even after 1 hr incubation at 1 mM glucose, yet they were further increased at 20 mM glucose (Figure 5C). Glucose also increased fumarate levels in CTL islets; 1 hr incubation at 20 mM glucose increased fumarate levels 12-fold, compared to islets incubated at 1 mM glucose. This was not due to a reduction in FH (Figure 5D) and, thus, is likely a consequence of the enhanced glucose metabolism. Similar acute (1 hr) effects of high glucose on fumarate content were observed in ND human islets (Figures 5E and 5F). Interestingly, the fumarate content of islets from donors with type 2 diabetes (T2D) was higher than that in ND islets, and high glucose produced no further increase (Figure 5F).

Tandem mass spectrometry (MS/MS) analysis of islets from stage II Fh1βKO mice islets detected succination of key cysteine residues in glyceraldehyde 3-phosphate dehydrogenase (GAPDH; residue C150) (Figures 5G and 5H), guanosine monophosphate reductase (GMPR; C186) (Figure S6A; Table S2), and Parkinson's disease (autosomal recessive, early onset) 7 (PARK 7/DJ-1; C106). Succination of PARK7/DJ-1 (C106) was also observed in islets from a human T2D donor (Figure S6B; Table S2).

Succination of residue C150 of GAPDH was increased by 270% in Fh1βKO islets (Figures 5G and 5H). Succination of GAPDH, detected in the gastrocnemius muscle of diabetic rats, has been shown to reduce enzyme activity (Blatnik et al., 2008). This might limit glucose flux through glycolysis, leading to accumulation of upstream glycolytic 3- and 6-carbon intermediates. We tested the possible functional consequences by culturing CTL islets for 24 hr in the presence of 10 mM of the triose D-glyceraldehyde. This resulted in the complete loss of GSIS and correlated with a slight increase in islet insulin content (Figures S7A and S7B).

Impact of *Fh1* Deletion on Cytosolic Calcium and pH

Links have been proposed between glucose and pH_i (Shepherd and Henquin, 1995). The loss of glucose- and Ca^{2+}-dependent electrical activity in stage III Fh1βKO β cells is predicted to cause an alteration in Ca^{2+} handling. Intracellular accumulation of fumarate, the anion of fumaric acid, may lower cytoplasmic pH (pH_i) and thereby compromise GSIS, effects compounded by the inhibition of GAPDH and accumulation of acidic trioses. Therefore, we performed parallel measurements of $[Ca^{2+}]_i$ and pH_i in stages I and III Fh1βKO β cells and CTL cells. Changes in $[Ca^{2+}]_i$ were similar in stage I CTL and Fh1βKO β cells (Figures 6A and 6B). Thus, glucose, glyceraldehyde, and high K^+-induced membrane depolarization increased $[Ca^{2+}]_i$. Tolbutamide had little additional stimulatory effect on $[Ca^{2+}]_i$ when tested in the presence of 20 mM glucose (cf. Figure 4E). Although basal pH_i was slightly lower in β cells from stage I Fh1βKO mice than in CTL cells (as expected from the elevated fumarate), the responses to glucose and glyceraldehyde were similar (Figures 6A, 6B, and 6E): glucose and glyceraldehyde lowered pH_i

glycine; Thr, threonine; Arg, arginine; Tau, taurine; Ala, alanine; Tyr, tyrosine; GABA, gamma aminobutyric acid; Val, valine; Phe, phenylalanine; Ile, isoleucine; Leu, leucine.

(E) Insulin secretion from islets from stage II Fh1βKO (gray) and CTL (black) islets (n = 4 experiments; n = 3 mice per genotype) in the presence of 70 mM KCl and 2 mM or 20 mM glucose and 5 mM dimethylglutamate (DMG), as indicated. **p < 0.01 versus 2 mM glucose in Fh1βKO; ***p < 0.0001 versus 2 mM glucose (CTL); §§p < 0.01 CTL versus Fh1βKO in 20 mM glucose.

(F) Insulin secretion in wild-type islets (n = 13 experiments; n = 3 mice) at 1 mM and 20 mM glucose with the addition of dimethyl fumarate (5 mM DMF). **p < 0.001 or better versus 1 mM glucose.

Error bars represent ± SEM. See also Figures S3 and S4 and Table S1.

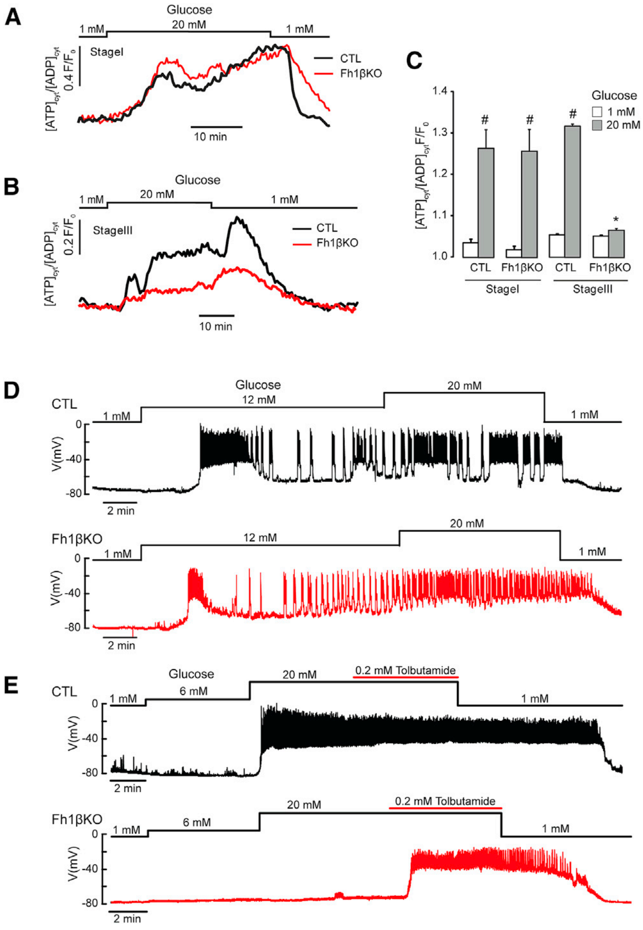

Figure 4. Fh1βKO β Cells Exhibit Impaired Electrical Activity and Reduced ATP Production with Age

(A) Glucose-induced changes in the ATP/ADP ratio in β cells from stage I CTL (black) and Fh1βKO (red) mice. Each trace is the average of >200 cells.

(B) Same as in (A) but using islets from stage III CTL and hyperglycemic Fh1βKO mice. Each trace is the average of >200 cells.

(C) Cytoplasmic ATP/ADP ratio at 1 mM and 20 mM glucose in islets from stage I and stage III Fh1βKO and age-matched CTL mice (n = 4 mice per genotype, >200 cells per mouse). Responses are normalized to the ratio at 1 mM glucose. *$p < 0.05$ versus CTL; #$p < 0.05$ versus basal (3 mM glucose). Error bars represent ± SEM.

(D) Glucose-induced electrical activity in β cells of stage II CTL or Fh1βKO β cells. Traces are representative of 5 (CTL) or 4 (Fh1βKO) β cells from at least 3 mice of each genotype.

(E) Same as in (D) but using islets from stage III CTL and Fh1βKO mice. Traces are representative of 5 (CTL) or 4 (Fh1βKO) β cells from at least 3 mice of each genotype.

reversibly in both CTL and Fh1βKO β cells, and high-$[K^+]_o$ depolarization produced a further drop in pH_i.

The $[Ca^{2+}]_i$ and pH_i responses of CTL β cells did not change with age, in contrast to the responses of those of stage III Fh1KβO mice (Figures 6C–6E). Both glucose and D-glyceraldehyde were without effect on $[Ca^{2+}]_I$, and tolbutamide was only effective in some β cells. However, high $[K^+]_o$ depolarization (which bypasses metabolism) consistently increased $[Ca^{2+}]_i$. Basal pH_i was lower in stage III Fh1βKO β cells than either CTL or stage I Fh1βKO β cells (Figures 6C–6E). Increasing glucose to 20 mM or the addition of 10 mM D-glyceraldehyde reduced pH_i in CTL β cells but did not cause further acidification in stage III Fh1βKO β cells (Figures 6D and 6E).

Impaired GSIS in Fh1βKO Islets Is a Consequence of Hyperglycemia

We reasoned that the deterioration of GSIS in Fh1βKO islets over time might be a consequence of the progressive hyperglycemia (Brereton et al., 2014). We tested whether the impairment of GSIS could be reversed. Freshly isolated islets from stage III Fh1βKO mice were refractory to 6 mM glucose, and the response to 20 mM glucose was only 30% of that seen in CTL islets (Figure 7A), echoing the data from the perfusion experiments (Figure 2B).

After culture at 12 mM glucose for ~65 hr, secretory responses in CTL islets were essentially the same as in freshly isolated islets. However, GSIS at 20 mM glucose was dramatically improved in Fh1βKO islets and approached that of the CTL islets (Figure 7B). Insulin content was 35% lower in Fh1βKO than in CTL islets (Figure 7C).

Finally, we tested whether the loss of GSIS in stage III Fh1βKO islets reflects a direct "glucotoxic" effect or is related to accelerated glucose metabolism. Distinct from what was seen following culture at 12 mM glucose (Figures 7A and 7B), islets from stage III Fh1βKO mice cultured for 3 days at 20 mM glucose showed no recovery of GSIS. By contrast, islets cultured in the presence of the glucokinase inhibitor mannoheptulose (Zelent et al., 2005; Coore and Randle, 1964) exhibited some (limited) glucose responsiveness (Figure 7D).

DISCUSSION

We show here that lack of the Krebs-cycle enzyme FH causes a progressive deterioration of β cell function, resulting in severe diabetes associated with impaired oxidative metabolism, ATP production, intracellular calcium handling, and cytosolic acidification.

Distinct from the mouse model in which *Vhl* is deleted in β cells (Zehetner et al., 2008; Cantley et al., 2009), we found that the diabetes associated with *Fh1* loss is *Hif1α* and *Nrf2* independent. Our data are consistent with earlier observations that, while *Fh1* deletion leads to the accumulation of fumarate, and HIF1α is stabilized, many associated functional changes are independent of HIF1α (O'Flaherty et al., 2010; Adam et al., 2013; Ternette et al., 2013). Similar to previous studies, we were unable to detect HIF1α in normal β cells, and deletion of *Hif1α* alone did not disrupt glucose homeostasis (Zehetner et al., 2008; Cantley et al., 2009). However, it remains unclear whether β cell dysfunction and diabetes are caused by HIF1α stabilization or vice versa (Cantley et al., 2010; Girgis et al., 2012). In the Fh1βKO mouse, loss of glucose regulation appears entirely attributable to FH. The hyperglycemia that develops may drive HIF1α stabilization, perhaps compounding GSIS impairment further in a feedback loop.

Why Do Young (Stage 1) Fh1βKO Mice Have Normal Glucose Tolerance?

Given the importance of mitochondrial metabolism for insulin secretion, the mild phenotype of stage I Fh1βKO mice was

OPEN
ACCESS
CellPress

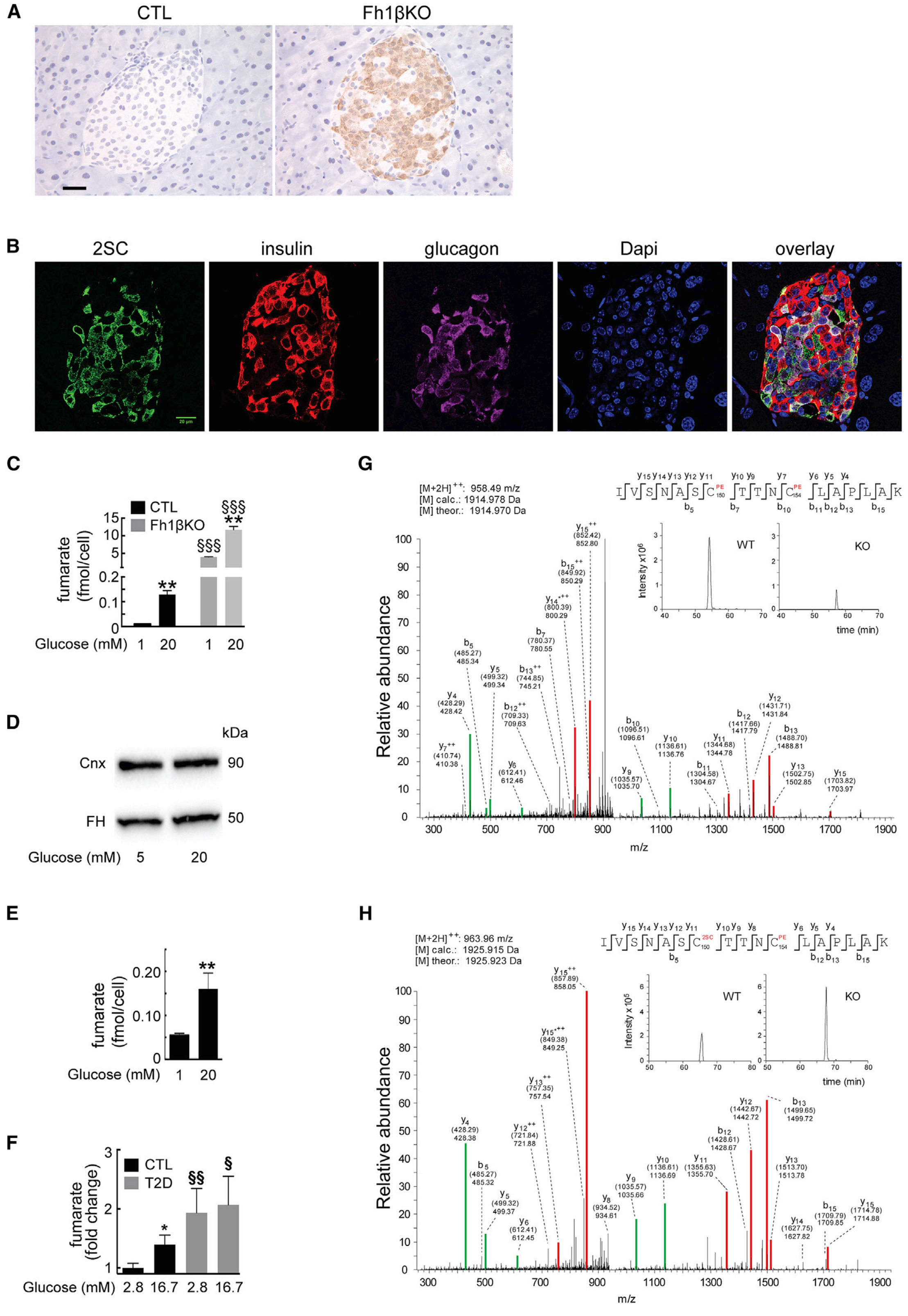

(legend on next page)

surprising, not least as *Fh1* deletion led to >100-fold accumulation of fumarate, consistent with arrest of the Krebs cycle at FH. Because insulin secretion was unimpaired, this suggests that refilling of Krebs-cycle intermediates (anaplerosis) must occur distal to FH. Labeling experiments in mouse embryonic fibroblasts (MEFs) lacking *Fh1* show that fumarate exits the mitochondria to the cytoplasm but is then metabolized to aspartate via the urea cycle, which re-enters the Krebs cycle (Adam et al., 2013). Studying metabolism in murine pancreatic islets is difficult because of the limited amounts of material available, especially from the diabetic islets. Our analyses of Fh1βKO islets by capillary electrophoresis time of flight MS (CE-TOFMS) indicate similar disruption of the urea cycle and purine metabolism and explain, in part, why the diabetic phenotype of Fh1βKO mice can be rescued by re-expression of cytosolic FH. Moreover, Fh1βKO islets exhibit increased utilization and oxidation of glucose, consistent with aerobic glycolysis, recapitulating previous observations made when *Fh1* was deleted in MEFs and mouse kidney (O'Flaherty et al., 2010; Adam et al., 2013).

Normally, excess Krebs-cycle reactants are used to produce amino acids via cataplerosis (Choi et al., 2011; MacDonald et al., 2005). However, in Fh1βKO islets, amino acid content tended to be reduced, and glucose did not increase the content of several amino acids; for example, glutamate. This is of interest because glutamate has been implicated in the amplification of GSIS (Maechler and Wollheim, 2000). Exogenous dimethyl glutamate restored GSIS in Fh1βKO islets but had no effect in CTL islets. These data support the idea that glutamate functions as an "amplification signal." Another possibility is that exogenous glutamate may restore GSIS by feeding into the Krebs cycle and partially restoring mitochondrial metabolism (Frezza et al., 2011).

Despite the ~50% reduction in insulin content, the response to 20 mM glucose was maintained in stage II Fh1βKO mice. We speculate that Fh1βKO β cells may compensate for the reduction of insulin content by stimulation of insulin exocytosis. Fh1βKO islets contain significantly elevated levels of adenylosuccinate, which has been proposed to stimulate exocytosis by inhibition of the sentrin/SUMO-specific protease 1 (Gooding et al., 2015).

Rapid Progression to Diabetes in Stage II Fh1βKO Mice

Once slight hyperglycemia developed, rapid deterioration of glycemic control was observed (up to 10 mM/week). We speculate that a small elevation of plasma glucose induces a vicious cycle of impaired insulin secretion and hyperglycemia. In part, this arises because, as blood glucose increases, more glucose enters metabolism, causing even greater stress on the β cell with increased fumarate levels. It is possible that metabolism of cytosolic fumarate and anaplerosis eventually become unable to maintain the Krebs cycle and that, once this occurs, rapid deterioration of both GSIS and glucose tolerance occurs.

Why Do Fh1βKO Mice Become Glucose Intolerant?

Our results indicate that Fh1βKO mice eventually develop diabetes because glucose is no longer able to stimulate insulin secretion. This is because of a failure of mitochondrial metabolism that culminates in impaired ATP production, defective K_{ATP}-channel closure, and suppression of electrical activity and $[Ca^{2+}]_i$.

Why does ATP production fail? Pumping H^+ across the inner mitochondrial membrane, the electron transport chain produces the alkalinization of the mitochondrial matrix (Wiederkehr et al., 2009) and membrane hyperpolarization, which are needed to drive ATP synthesis in β cells. Fumaric acid ($C_4H_4O_4$) is an acid with two hydroxyl groups with pKa of 3.0 and 4.4; thus, loss of FH activity can accordingly be expected to result in acidification not just of the cytoplasm (experimentally measured) but also of the mitochondrial matrix.

There is a sigmoidal relationship between glucose concentration and mitochondrial metabolism (Ashcroft et al., 1970; Hellman et al., 1971). At low glucose concentrations, the Krebs cycle runs at a fairly low rate, and the amount of fumarate generated and deposited in the cytosol will be low. Thus, if Fh1βKO mice remain normoglycemic, cytoplasmic acidification will be modest. In agreement with this idea, glucose-responsive β-cells

Figure 5. Succination Is a Feature of Elevated Fumarate

(A) IHC for 2SC in islets from stage II CTL (left) and Fh1βKO (right) islets (n > 300 islets from 10 mice of each genotype). Scale bar, 20 μm.

(B) Immunofluorescence for 2SC (green), insulin (red), glucagon (purple), nuclei (blue), and overlay in a representative islet from a stage III Fh1βKO mouse (n > 100 islets from n > 10 Fh1βKO mice). Scale bar, 20 μm.

(C) Fumarate content determined by CE-TOFMS in islets from stage II Fh1βKO mice (gray; all islets from n = 6 mice) and CTL mice (black; all islets from n = 6 mice) incubated for 1 hr at 1 mM or 20 mM glucose. **p < 0.01 versus 1 mM glucose; §§§p < 0.01 versus CTL.

(D) Western blot of FH protein in islets from stage II CTL mice (all islets from n = 3 mice) cultured for 24 hr at 5 mM or 20 mM glucose. Cnx, calnexin, loading control.

(E) Fumarate content measured by CE-TOFMS of islets from human ND donors (4 donors) incubated for 1 hr at 1 mM or 20 mM glucose. **p < 0.01 versus 1 mM glucose.

(F) Fumarate content determined by gas chromatography-mass spectrometry (GC-MS) in human ND (31 donors) or T2D (7 donors) islets cultured at 2.8 mM and 16.7 mM glucose. Content is expressed relative to that of ND islets at 2.8 mM glucose.

(G and H) MS/MS spectra for the GAPDH-derived peptide IVSNASCTTNCLAPLAK in its non-succinated (G) and succinated (H) forms in stage II Fh1βKO and CTL islets (at least 150 islets per genotype). The calculated peptide mass based on the detected m/z (m, mass; z, charge) value of the doubly charged precursor peptide ion ($[M+2H]^{2+}$) and the calculated ([M] calc.) and theoretical peptide mass ([M] theor.) are stated for both peptide species. Detected N- and C-terminal fragment ions are indicated in the peptide sequence, assigned in the spectrum and depicted as follows: b: N-terminal fragment ion; y: C-terminal fragment ion; *: fragment ion minus NH_3; and $^{2+}$: doubly charged fragment ion. Both theoretical mass (in brackets) and detected mass are given for each assigned fragment ion. Peptide fragments that include the succinated cysteine residue are highlighted in red, while unsuccinated fragments are depicted in green.

Insets in (G) and (H) show the extracted ion chromatograms of the precursor peptide from a representative triplicate run of analyzed pancreatic islets. Quantification of the peptide, corresponding to residues 144–160 of Gapdh, succinated at C150, indicate that the succinated peptide was enriched by 270%, while the unmodified peptide was decreased by 70%. PE indicates the modification of cysteine residues at C150 and C154 to pyridylethyl-cysteine in the inset in (G) and only C154 in (H). Error bars represent ± SEM. See also Figure S6 and Table S2.

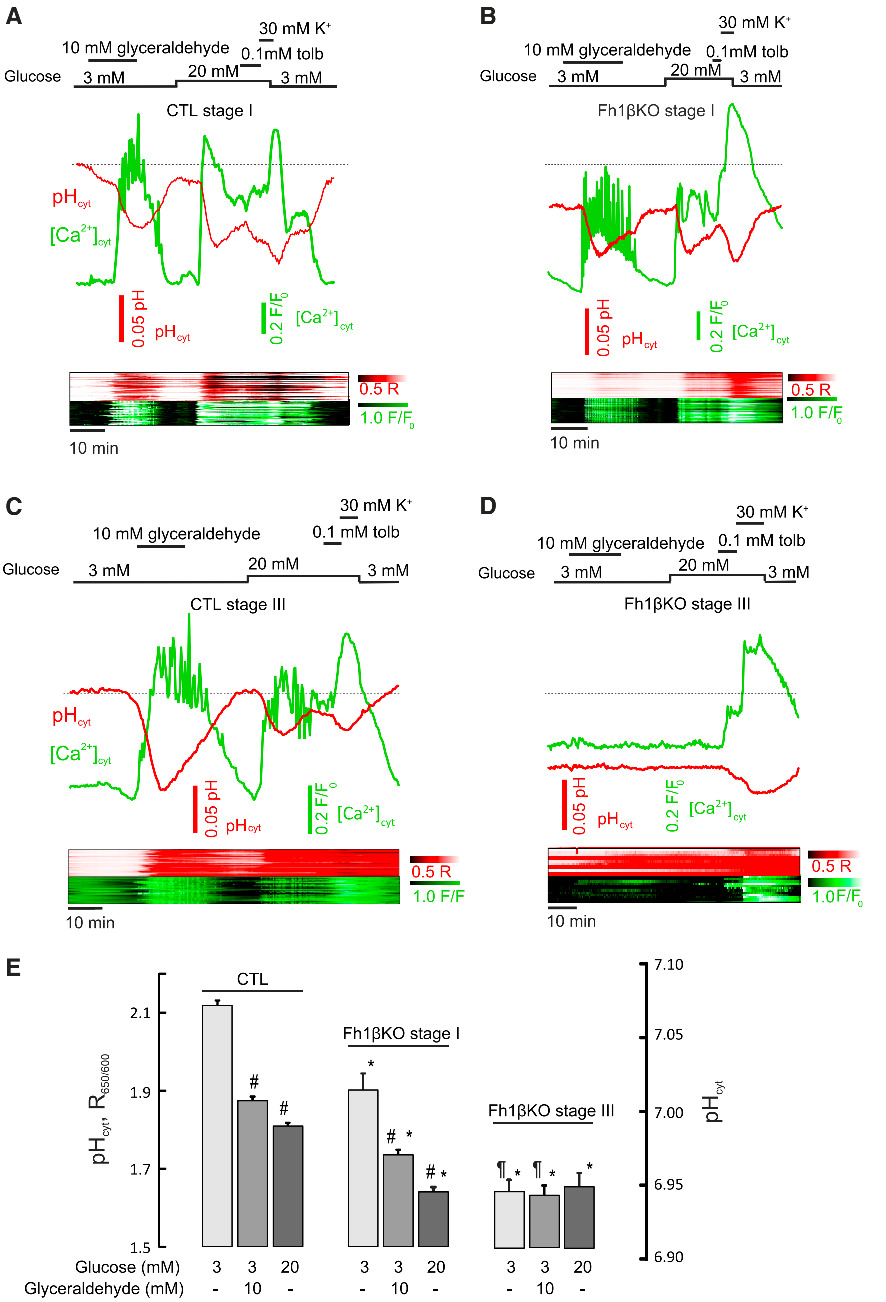

Figure 6. Effect of *Fh1* Deletion on $[Ca^{2+}]_i$ and pH_i in β Cells

(A and B) Top: simultaneous measurements of $[Ca^{2+}]_i$ (green) and pH_i (red) in β-cells within intact pancreatic islets from stage I CTL (A) and Fh1βKO (B) mice exposed to 3 mM or 20 mM glucose, 10 mM D-glyceraldehyde, 30 mM K^+, or 0.1 mM tolbutamide. The dashed line indicates basal pH_i. Bottom: heatmaps showing pH_i (red) and $[Ca^{2+}]_i$ (green) responses for individual cells (>20 cells within a single islet). Color intensity indicates concentration range from low (black) to high (white). See calibration bars at right.

(C and D) Same as in (A) and (B) but using islets from stage III CTL (C) and Fh1βKO (D) mice.

(E) Mean ± SEM fluorescence ratio (left) at 3 mM glucose, with or without 10 mM D-glyceraldehyde or 20 mM glucose in β cells from stage I (gray bars) Fh1βKO (n = 95 cells from 2 mice) and stage III (white bars) Fh1βKO (n = 39 cells from 4 mice) mouse islets. $^{\#}p < 0.05$ versus basal (3 mM) glucose and CTL (n = 163 cells from 6 mice; there was no difference between stage I and stage III CTL β cells, and data have been pooled for display). $^{*}p < 0.05$ versus CTL; $^{¶}p < 0.05$ versus stage I Fh1βKO β cells. Approximate changes in calibrated pH_i are shown (right).

from stage I Fh1βKO mice had a higher pH_i than β cells from stage III mice. At higher plasma glucose levels, mitochondrial glucose metabolism will accelerate due to the increased substrate, leading to greater fumarate generation, acidification of both cytoplasmic and mitochondrial matrices, and a progressive impairment of ATP production. Also, acidification may influence the activity of other enzymes involved in β cell metabolism with optimal activity at alkaline pH (e.g., Bernstein and Everse, 1978; Lai and Cooper, 1986; Willson and Tipton, 1980). These effects may be aggravated by downregulation of key genes involved in mitochondrial metabolism, as seen in a mouse model of diabetes caused by a gain-of-function mutation in the K_{ATP} channel (βV59M mice; Brereton et al., 2016). These scenarios are not mutually exclusive, and it is possible that they operate in parallel and that both contribute to the loss of ATP production and GSIS. Both these hypotheses predict that inhibition of glucose metabolism should exert a protective effect on β cell metabolism. This proved to be the case, as GSIS was restored in Fh1βKO islets following "normoglycemic" culture or culture in high glucose with the glucokinase inhibitor mannoheptulose.

Fumarate Accumulation Leads to Protein Succination

Our data demonstrate that elevation of intracellular fumarate is associated with "hyperglycemia" in both mouse and human islets. We detected succination of critical cysteines in GAPDH, GMPR, and PARK7/DJ-1 proteins in Fh1βKO islets. Succination of GAPDH has been reported previously in adipocytes in diabetic *db/db* and *ob/ob* mice. It is a marker of impaired mitochondrial metabolism and has functional effects (Blatnik et al., 2008; Frizzell et al., 2011). Reduced PARK7/DJ1 activity is compatible with the small mitochondria (suggestive of mitochondrial fragmentation and impaired function) seen in Fh1βKO islets. Interestingly, we found that fumarate levels are elevated in islets from T2D donors and may, via succination, explain why expression of PARK7 is

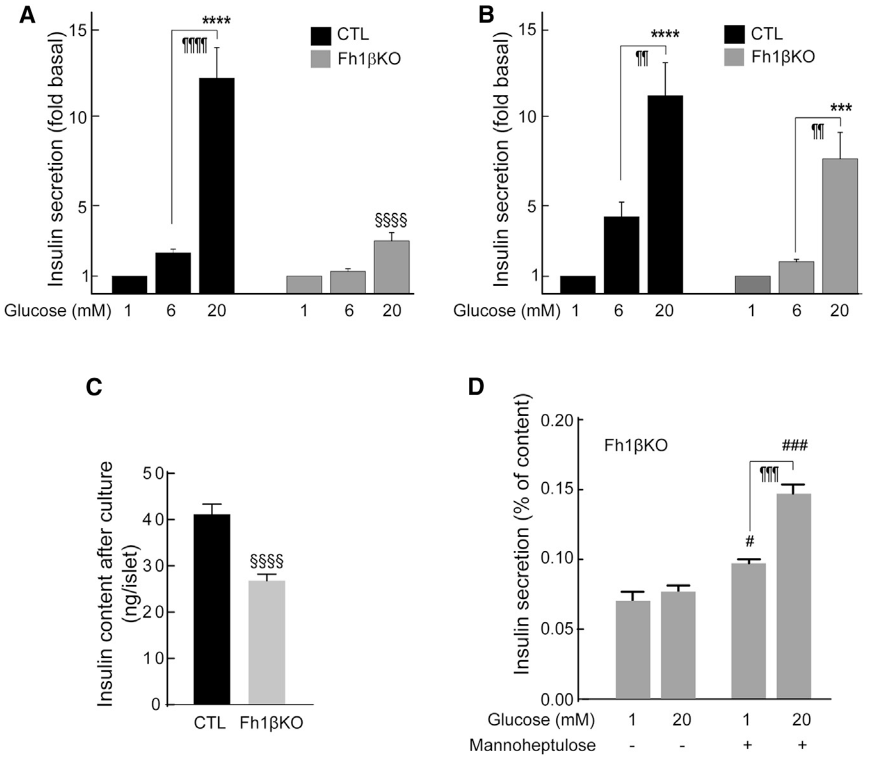

Figure 7. GSIS Dysfunction in Diabetic Fh1βKO Islets Can Be Reversed by "Normoglycemia"

(A) Insulin secretion from freshly isolated islets from stage III Fh1βKO (gray) and CTL (black) littermates at 1 mM, 6 mM, or 20 mM glucose during static incubation (n = 12–18 experimental groups of islets from at least 8 mice of each genotype in a total of 3 experiments). ****$p < 0.0001$ versus 1 mM glucose; ¶¶¶¶$p < 0.0001$ versus 6 mM glucose; §§§§$p < 0.0001$ versus 20 mM glucose in CTL.

(B) Insulin secretion from the same islets as in (A) after 65-hr culture at 12 mM glucose. ***$p < 0.001$ versus 1 mM glucose; ¶¶$p < 0.01$ versus 6 mM glucose; §§§§$p < 0.0001$ versus 20 mM glucose in CTL.

(C) Insulin content of islets used in (B). §§§§$p < 0.0001$.

(D) Insulin secretion in islets isolated from stage III Fh1βKO mice measured in static incubations at 1 mM or 20 mM glucose after 72-hr culture at 20 mM glucose with or without 10 mM mannoheptulose (n = 3 mice). ¶¶¶$p < 0.001$ versus 1 mM glucose; #$p < 0.05$, and ###$p < 0.001$ versus corresponding condition without prior culture in the presence of mannoheptulose.

Error bars represent ± SEM. See also Figure S7.

reduced in T2D islets (Jain et al., 2012). Importantly, it also suggests that mitochondrial metabolism is impaired in islets, leading to elevated fumarate and succination.

Conclusions

Our studies suggest a cycle in which progressive hyperglycemia in Fh1βKO mice leads to the deterioration of metabolism, culminating in the loss of GSIS and frank diabetes. Although β cells from young Fh1βKO mice are glucose responsive, subtle differences exist between Fh1βKO and CTL islets. Thus, GSIS is impaired at 6 mM glucose in Fh1βKO mice, compared to CTL mice. This defect may underlie the slight elevation in plasma glucose that precedes the more rapid deterioration in glucose tolerance. Our hypothesis is consistent with the proposal that hyperglycemia, via β cell decompensation, initiates a cycle of progressive hyperglycemia and impaired GSIS (Weir and Bonner-Weir, 2004).

In severely diabetic mice expressing the gain-of-function K_{ATP} channel mutation V59M (Brereton et al., 2014), the adverse effects on β cell function and insulin content were reversed following restoration of normoglycemia. Similarly, the severe impairment of GSIS seen in diabetic (stage III) Fh1βKO islets was almost fully corrected simply by culturing the islets under "normoglycemic" conditions. This agrees with the report that GSIS is restored in long-term T2D patients following normalization of plasma glucose levels and suppression of hepatic glucose production induced by a low-calorie diet (Lim et al., 2011).

We propose that the Fh1βKO mouse provides a valuable new model for T2D. In particular, it is not obese, and the glucose intolerance develops in an age-dependent fashion. Thus, it provides a useful tool for studying the progression observed in T2D and to interrogate the systemic and cellular consequences of metabolic dysfunction in the pancreatic β cell without the complications of altered diet and/or obesity.

EXPERIMENTAL PROCEDURES

See also Supplemental Experimental Procedures.

Mice

Animal experiments were conducted in accordance with the UK Animals Scientific Procedures Act (1986) and University of Oxford local ethical guidelines. We used male and female adult mice of the following strains: *$Fh1^{tm1Pjpfl/fl}$Rip2-$Cre^{+/-}$* (*$Fh1^{tm1Pjpfl/fl}$* crossed with Tg(*Ins2-Cre*)^{23Herr}Cre recombinase, *Rip2-$Cre^{+/-}$*), *$Fh1^{tm1Pjpfl/fl}$$Hif1\alpha^{fl/fl}$Rip2-$Cre^{+/-}$*, *$Fh1^{tm1Pjpfl/fl}$Rip2-$Cre^{+/-}$Gt(ROSA) $26Sor^{tm1(CAG-FH)Pjp}$;Tg(Cdh16-cre)91Igr*, *$Fh1^{tm1Pjp}$Gt(ROSA)$26Sor^{tm1(CAG-FH')Pjp}$ Tg(Cdh16-cre)91Igr*, and *$Fh1^{tm1Pjpfl/fl}$Rip2-$Cre^{+/-}$$Nrf2^{-/-}$* mice (designated Fh1βKO, Fh1Hif1αβKO, Fh1βKO+FH, Fh1βKO+FH^{cyt}, and Fh1β/Nrf2KO, respectively) and littermate controls (designated CTL). The constitutive Nrf2 KO mouse was generated from an embryonic stem cell (ESC) clone obtained from Riken, Japan. Genotyping was performed by Transnetyx, but primer details and PCR conditions can be obtained from J.A. NMRI and C57BL/6J mice (designated wild-type) purchased commercially were used in a few cases.

IHC

Mouse tissues were fixed in 10% neutral-buffered formalin, dehydrated, and processed for paraffin wax embedding and sectioning (3 μm). H&E sections were generated for all samples. IHC was carried out using the EnVision Kit (Dako) as per the manufacturer's protocol, with the following antibodies: FH (Autogen Bioclear), HIF1α (Cayman), insulin (MP Biomedicals), glucagon (MP Biomedicals and Sigma), and 2SC (Nagai et al., 2007).

Intraperitoneal Glucose Tolerance Test

Blood glucose levels were determined with an Accuchek Aviva meter after 16 hr of fasting and at time points following intraperitoneal injection of 2 g glucose per kilogram of body weight. Animals were culled at the end of the test, and pancreata were processed as described earlier.

Hormone Secretion and Content Measurements

Insulin secretion was measured by in situ pancreas perfusion or in static incubations of isolated islets (Zhang et al., 2013). Insulin content was determined in parallel from isolated islets or from mouse pancreata harvested separately.

Quantitative Imaging of ATP, Ca^{2+}, and pH_i

Time-lapse imaging of the ATP/ADP ratio in mouse islets was performed using 10×–14× magnification on a Zeiss AxioZoom.V16 microscope. Islets were infected with an adenovirus (3 × 10^4 plaque-forming units [PFUs] per islet) delivering Perceval, a recombinant sensor of ATP/ADP (Berg et al., 2009). Groups of islets isolated from CTL and Fh1βKO animals were imaged simultaneously 24 hr post-infection at glucose concentrations as indicated, with single-cell resolution. Time-lapse images were collected every 30 s, and the bath solution was perifused at 60 μL/min at 34°C.

Simultaneous time-lapse imaging of $[Ca^{2+}]_i$ and pH_i in mouse islets was performed on the inverted Zeiss AxioVert 200 microscope, equipped with the Zeiss LSM 510-META laser confocal scanning system, using a 40×/1.3 NA objective. Mouse islets were loaded with 6 μM of the Ca^{2+}-sensitive dye Fluo-4 for 90 min before being transferred to a separate solution containing 6 μM of the pH-sensitive dye SNARF-5F (both dyes from Molecular Probes) for a further 50 min at room temperature and imaged using an open chamber at 34°C. The bath solution containing various stimuli was perifused continuously at the rate of 200 μL/min. The ratiometric dye SNARF-5F was excited at 543 nm, and emission was collected at 650 nm and 600 nm. Fluo-4 was excited at 488 nm and imaged at 530 nm. Images were acquired at the frequency of 0.03 Hz. The pH calibration of each trace was performed using the high K^+-nigericin technique. Valinomycin (5 μM) was added to the extracellular solution to abolish the K^+ gradient (Tarasov et al., 2012, 2013).

Statistical Analysis

Image sequences were analyzed (registration, background subtraction, ROI intensity versus time analysis) using open-source FIJI software (http://fiji.sc/Fiji). The numerical time series data were analyzed using the IgorPro package (Wavemetrics). Statistical significance of the differences between paired or unpaired samples was tested using Friedman or Kruskall-Wallis tests, respectively, with Nemenyi post hoc analysis, as implemented in the R package (R Development Core Team, 2016). Basal pH_i was calculated by taking the mean of the first 15 SNARF-5F ratio values from each cell. After glyceraldehyde and 20 mM glucose application, pH_i was calculated by taking a mean of the values when the SNARF-5F ratio reached a nadir. Differences with $p < 0.05$ were considered significant. Cells that were not active at 3 mM glucose and that responded to high glucose with the characteristic $[Ca^{2+}]_i$ oscillations were taken as β cells.

All data are given as mean ± SEM unless indicated that they are mean ± SD. Other than for image analysis, as indicated earlier, statistical significance was determined with significance set at <0.05, using either ANOVA with Tukey's multiple comparison or Student's t test (where indicated). Statistical significance was determined using GraphPad Prism v.6.0d (GraphPad Software, La Jolla, CA, USA; http://www.graphpad.com).

Human Islets and Ethics

Pancreatic islets were isolated from deceased donors under ethical approval obtained from the local human research ethics committees in both Oxford and Lund. All donors gave informed research consent. Islets were obtained from the Diabetes Research & Wellness Foundation Human Islet Isolation Facility, OCDEM, University of Oxford, and the Nordic Center for Clinical Islet Transplantation (http://www.nordicislets.com; Uppsala, Sweden) via the Human Tissue Laboratory at Lund University Diabetes Centre. Islets were hand picked, and their quality was assessed prior to research use. Experiments in Oxford were performed using islets from donors (5 females and 1 male) with the following parameters: age, 47.8 years ± 6.7 years; BMI, 28.4 ± 5.8. In Lund, the characteristics of ND (n = 31, CTL) and diabetic (n = 7, T2D) islet donors were as follows: age (years), 61.24 ± 10.45 (CTL) and 60.43 ± 7.72 (T2D), p = 0.82; sex, expressed as male/female, 14/7 (CTL) and 2/5 (T2D), p = 0.44; BMI, 27.11 ± 3.06 (CTL) and 28.9 ± 5.35 (T2D), p = 0.42; and HbA1c, 5.85 ± 0.45 (CTL) and 6.41 ± 0.56, p = 0.04. Data are given as mean ± SD; groups were compared using the Student's t test or using two-tailed Fisher's exact test.

SUPPLEMENTAL INFORMATION

Supplemental Information includes Supplemental Experimental Procedures, seven figures, and two tables and can be found with this article online at http://dx.doi.org/10.1016/j.celrep.2017.08.093.

AUTHOR CONTRIBUTIONS

J.A., P.J.P., and P.R. designed the study. J.A., R.R., M.V.C., N.T., A.H., A.I.T., Q.Z., E.R., N.J.G.R., R.M.d.R., G.O., H.W.D., P.S., K.S., K.K., K.I., B.M.K., J.T.-R., H.M., A.C., T.S., and P.J.P. collected and analyzed the data. A.L., A.S., C.W.P., N.F., and P.R. analyzed data and contributed expertise. P.J.P., M.V.C., A.S., A.I.T., and J.A. made the final figures. J.A., A.S., P.J.P., P.R., and F.M.A. wrote the manuscript, which was critically edited by all authors.

ACKNOWLEDGMENTS

We thank David Wiggins for technical support, staff at the Oxford University Biomedical Services facilities for taking care of the mice, and Professor P.R.V. Johnson and his team for provision of human pancreatic islets. We are grateful to Randall Johnson and Pedro Herrera for providing Hif1α and Rip-Cre mice, respectively, and Ralph Deberardinis for critical evaluation of the metabolomics analyses. We thank the Wellcome Trust for core infrastructure support to the Wellcome Trust Centre for Human Genetics, Oxford (090532/Z/09/Z), a Wellcome Trust Senior Investigator Award (095531/Z/11/Z) to P.R., and grants to F.M.A. (884655 and 089795). T.S. is supported by a Grant-in-Aid for Scientific Research on Innovative Areas, Japan (22134007) and the Yamagata Prefectural Government and City of Tsuruoka; H.M., P.S., and P.R. are supported by the Swedish Research Council; P.R. is supported by the Knut and Alice Wallenbergs Stiftelse (Wallenberg Scholars); and E.R. is supported by FAPESP. R.M.d.R. and J.T.-R. were partially supported by a grant from the Spanish Ministry of Science and Technology: SAF2009/1267. F.M.A. holds a Royal Society/Wolfson merit award and an ERC Advanced Investigatorship (322620). A.L. is a Barts and The London Charity post-doctoral fellow. R.R. and Q.Z. are Diabetes UK RD Lawrence fellows, and A.H. holds a PhD studentship funded by Diabetes UK.

This paper is a tribute to Dr. Patrick John Pollard, who died unexpectedly in June 2015. He was an enthusiastic and exceptionally gifted scientist. We remember him fondly as a friend and colleague who made important contributions, not only to this study, but also to the field of cancer metabolism.

Received: May 26, 2015
Revised: July 27, 2017
Accepted: August 29, 2017
Published: September 26, 2017

REFERENCES

Adam, J., Hatipoglu, E., O'Flaherty, L., Ternette, N., Sahgal, N., Lockstone, H., Baban, D., Nye, E., Stamp, G.W., Wolhuter, K., et al. (2011). Renal cyst formation in Fh1-deficient mice is independent of the Hif/Phd pathway: roles for fumarate in KEAP1 succination and Nrf2 signaling. Cancer Cell *20*, 524–537.

Adam, J., Yang, M., Bauerschmidt, C., Kitagawa, M., O'Flaherty, L., Maheswaran, P., Özkan, G., Sahgal, N., Baban, D., Kato, K., et al. (2013). A role for cytosolic fumarate hydratase in urea cycle metabolism and renal neoplasia. Cell Rep. *3*, 1440–1448.

Adam, J., Yang, M., Soga, T., and Pollard, P.J. (2014). Rare insights into cancer biology. Oncogene *33*, 2547–2556.

Alderson, N.L., Wang, Y., Blatnik, M., Frizzell, N., Walla, M.D., Lyons, T.J., Alt, N., Carson, J.A., Nagai, R., Thorpe, S.R., and Baynes, J.W. (2006). S-(2-Succinyl)cysteine: a novel chemical modification of tissue proteins by a Krebs cycle intermediate. Arch. Biochem. Biophys. *450*, 1–8.

Ashcroft, F.M., and Rorsman, P. (2012). Diabetes mellitus and the β cell: the last ten years. Cell *148*, 1160–1171.

Ashcroft, S.J., Hedeskov, C.J., and Randle, P.J. (1970). Glucose metabolism in mouse pancreatic islets. Biochem. J. *118*, 143–154.

Berg, J., Hung, Y.P., and Yellen, G. (2009). A genetically encoded fluorescent reporter of ATP:ADP ratio. Nat. Methods *6*, 161–166.

Bernstein, L.H., and Everse, J. (1978). Studies on the mechanism of the malate dehydrogenase reaction. J. Biol. Chem. *253*, 8702–8707.

Blatnik, M., Frizzell, N., Thorpe, S.R., and Baynes, J.W. (2008). Inactivation of glyceraldehyde-3-phosphate dehydrogenase by fumarate in diabetes: formation of S-(2-succinyl)cysteine, a novel chemical modification of protein and possible biomarker of mitochondrial stress. Diabetes *57*, 41–49.

Bond, M.R., and Hanover, J.A. (2013). O-GlcNAc cycling: a link between metabolism and chronic disease. Annu. Rev. Nutr. *33*, 205–229.

Brereton, M.F., Iberl, M., Shimomura, K., Zhang, Q., Adriaenssens, A.E., Proks, P., Spiliotis, I.I., Dace, W., Mattis, K.K., Ramracheya, R., et al. (2014). Reversible changes in pancreatic islet structure and function produced by elevated blood glucose. Nat. Commun. *5*, 4639.

Brereton, M.F., Rohm, M., Shimomura, K., Holland, C., Tornovsky-Babeay, S., Dadon, D., Iberl, M., Chibalina, M.V., Lee, S., Glaser, B., et al. (2016). Hyperglycaemia induces metabolic dysfunction and glycogen accumulation in pancreatic β-cells. Nat. Commun. *7*, 13496.

Bulusu, V., Jayaraman, V., and Balaram, H. (2011). Metabolic fate of fumarate, a side product of the purine salvage pathway in the intraerythrocytic stages of *Plasmodium falciparum*. J. Biol. Chem. *286*, 9236–9245.

Cantley, J., Selman, C., Shukla, D., Abramov, A.Y., Forstreuter, F., Esteban, M.A., Claret, M., Lingard, S.J., Clements, M., Harten, S.K., et al. (2009). Deletion of the von Hippel-Lindau gene in pancreatic β cells impairs glucose homeostasis in mice. J. Clin. Invest. *119*, 125–135.

Cantley, J., Grey, S.T., Maxwell, P.H., and Withers, D.J. (2010). The hypoxia response pathway and β-cell function. Diabetes Obes. Metab. *12* (*Suppl. 2*), 159–167.

Choi, S.E., Lee, Y.J., Hwang, G.S., Chung, J.H., Lee, S.J., Lee, J.H., Han, S.J., Kim, H.J., Lee, K.W., Kim, Y., et al. (2011). Supplement of TCA cycle intermediates protects against high glucose/palmitate-induced INS-1 beta cell death. Arch. Biochem. Biophys. *505*, 231–241.

Coore, H.G., and Randle, P.J. (1964). Inhibition of glucose phosphorylation by mannoheptulose. Biochem. J. *91*, 56–59.

Cramer, T., Yamanishi, Y., Clausen, B.E., Förster, I., Pawlinski, R., Mackman, N., Haase, V.H., Jaenisch, R., Corr, M., Nizet, V., et al. (2003). HIF-1alpha is essential for myeloid cell-mediated inflammation. Cell *112*, 645–657.

Frezza, C., Zheng, L., Folger, O., Rajagopalan, K.N., MacKenzie, E.D., Jerby, L., Micaroni, M., Chaneton, B., Adam, J., Hedley, A., et al. (2011). Haem oxygenase is synthetically lethal with the tumour suppressor fumarate hydratase. Nature *477*, 225–228.

Frizzell, N., Lima, M., and Baynes, J.W. (2011). Succination of proteins in diabetes. Free Radic. Res. *45*, 101–109.

Girgis, C.M., Cheng, K., Scott, C.H., and Gunton, J.E. (2012). Novel links between HIFs, type 2 diabetes, and metabolic syndrome. Trends Endocrinol. Metab. *23*, 372–380.

Gooding, J.R., Jensen, M.V., Dai, X., Wenner, B.R., Lu, D., Arumugam, R., Ferdaoussi, M., MacDonald, P.E., and Newgard, C.B. (2015). Adenylosuccinate is an insulin secretagogue derived from glucose-induced purine metabolism. Cell Rep. *13*, 157–167.

Hellman, B., Sehlin, J., and Täljedal, I.B. (1971). Effects of glucose and other modifiers of insulin release on the oxidative metabolism of amino acids in micro-dissected pancreatic islets. Biochem. J. *123*, 513–521.

Henquin, J.C. (2011). The dual control of insulin secretion by glucose involves triggering and amplifying pathways in β-cells. Diabetes Res. Clin. Pract. *93* (*Suppl. 1*), S27–S31.

Herrera, P.L. (2000). Adult insulin- and glucagon-producing cells differentiate from two independent cell lineages. Development *127*, 2317–2322.

Isaacs, J.S., Jung, Y.J., Mole, D.R., Lee, S., Torres-Cabala, C., Chung, Y.L., Merino, M., Trepel, J., Zbar, B., Toro, J., et al. (2005). HIF overexpression correlates with biallelic loss of fumarate hydratase in renal cancer: novel role of fumarate in regulation of HIF stability. Cancer Cell *8*, 143–153.

Itoh, K., Chiba, T., Takahashi, S., Ishii, T., Igarashi, K., Katoh, Y., Oyake, T., Hayashi, N., Satoh, K., Hatayama, I., et al. (1997). An Nrf2/small Maf heterodimer mediates the induction of phase II detoxifying enzyme genes through antioxidant response elements. Biochem. Biophys. Res. Commun. *236*, 313–322.

Jain, D., Jain, R., Eberhard, D., Eglinger, J., Bugliani, M., Piemonti, L., Marchetti, P., and Lammert, E. (2012). Age- and diet-dependent requirement of DJ-1 for glucose homeostasis in mice with implications for human type 2 diabetes. J. Mol. Cell Biol. *4*, 221–230.

Lai, J.C., and Cooper, A.J. (1986). Brain alpha-ketoglutarate dehydrogenase complex: kinetic properties, regional distribution, and effects of inhibitors. J. Neurochem. *47*, 1376–1386.

Launonen, V., Vierimaa, O., Kiuru, M., Isola, J., Roth, S., Pukkala, E., Sistonen, P., Herva, R., and Aaltonen, L.A. (2001). Inherited susceptibility to uterine leiomyomas and renal cell cancer. Proc. Natl. Acad. Sci. USA *98*, 3387–3392.

Lim, E.L., Hollingsworth, K.G., Aribisala, B.S., Chen, M.J., Mathers, J.C., and Taylor, R. (2011). Reversal of type 2 diabetes: normalisation of beta cell function in association with decreased pancreas and liver triacylglycerol. Diabetologia *54*, 2506–2514.

MacDonald, M.J., Fahien, L.A., Brown, L.J., Hasan, N.M., Buss, J.D., and Kendrick, M.A. (2005). Perspective: emerging evidence for signaling roles of mitochondrial anaplerotic products in insulin secretion. Am. J. Physiol. Endocrinol. Metab. *288*, E1–E15.

Maechler, P., and Wollheim, C.B. (1999). Mitochondrial glutamate acts as a messenger in glucose-induced insulin exocytosis. Nature *402*, 685–689.

Maechler, P., and Wollheim, C.B. (2000). Mitochondrial signals in glucose-stimulated insulin secretion in the beta cell. J. Physiol. *529*, 49–56.

Merkley, E.D., Metz, T.O., Smith, R.D., Baynes, J.W., and Frizzell, N. (2014). The succinated proteome. Mass Spectrom. Rev. *33*, 98–109.

Nagai, R., Brock, J.W., Blatnik, M., Baatz, J.E., Bethard, J., Walla, M.D., Thorpe, S.R., Baynes, J.W., and Frizzell, N. (2007). Succination of protein thiols during adipocyte maturation: a biomarker of mitochondrial stress. J. Biol. Chem. *282*, 34219–34228.

O'Flaherty, L., Adam, J., Heather, L.C., Zhdanov, A.V., Chung, Y.L., Miranda, M.X., Croft, J., Olpin, S., Clarke, K., Pugh, C.W., et al. (2010). Dysregulation of hypoxia pathways in fumarate hydratase-deficient cells is independent of defective mitochondrial metabolism. Hum. Mol. Genet. *19*, 3844–3851.

Pollard, P.J., Brière, J.J., Alam, N.A., Barwell, J., Barclay, E., Wortham, N.C., Hunt, T., Mitchell, M., Olpin, S., Moat, S.J., et al. (2005). Accumulation of Krebs cycle intermediates and over-expression of HIF1alpha in tumours which result from germline FH and SDH mutations. Hum. Mol. Genet. *14*, 2231–2239.

Pollard, P.J., Spencer-Dene, B., Shukla, D., Howarth, K., Nye, E., El-Bahrawy, M., Deheragoda, M., Joannou, M., McDonald, S., Martin, A., et al. (2007). Targeted inactivation of *fh1* causes proliferative renal cyst development and activation of the hypoxia pathway. Cancer Cell *11*, 311–319.

R Development Core Team (2016). R: A language and environment for statistical computing (R Foundation for Statistical Computing). http://www.R-project.org/.

Shepherd, R.M., and Henquin, J.C. (1995). The role of metabolism, cytoplasmic Ca2+, and pH-regulating exchangers in glucose-induced rise of cytoplasmic pH in normal mouse pancreatic islets. J. Biol. Chem. *270*, 7915–7921.

Spégel, P., Malmgren, S., Sharoyko, V.V., Newsholme, P., Koeck, T., and Mulder, H. (2011). Metabolomic analyses reveal profound differences in

glycolytic and tricarboxylic acid cycle metabolism in glucose-responsive and -unresponsive clonal β-cell lines. Biochem. J. *435*, 277–284.

Sullivan, L.B., Martinez-Garcia, E., Nguyen, H., Mullen, A.R., Dufour, E., Sudarshan, S., Licht, J.D., Deberardinis, R.J., and Chandel, N.S. (2013). The protooncometabolite fumarate binds glutathione to amplify ROS-dependent signaling. Mol. Cell *51*, 236–248.

Tarasov, A.I., Semplici, F., Ravier, M.A., Bellomo, E.A., Pullen, T.J., Gilon, P., Sekler, I., Rizzuto, R., and Rutter, G.A. (2012). The mitochondrial Ca2+ uniporter MCU is essential for glucose-induced ATP increases in pancreatic β-cells. PLoS ONE *7*, e39722.

Tarasov, A.I., Semplici, F., Li, D., Rizzuto, R., Ravier, M.A., Gilon, P., and Rutter, G.A. (2013). Frequency-dependent mitochondrial Ca(2+) accumulation regulates ATP synthesis in pancreatic β cells. Pflugers Arch. *465*, 543–554.

Ternette, N., Yang, M., Laroyia, M., Kitagawa, M., O'Flaherty, L., Wolhulter, K., Igarashi, K., Saito, K., Kato, K., Fischer, R., et al. (2013). Inhibition of mitochondrial aconitase by succination in fumarate hydratase deficiency. Cell Rep. *3*, 689–700.

Thomas, S.A., Storey, K.B., Baynes, J.W., and Frizzell, N. (2012). Tissue distribution of S-(2-succino)cysteine (2SC), a biomarker of mitochondrial stress in obesity and diabetes. Obesity (Silver Spring) *20*, 263–269.

Uruno, A., Furusawa, Y., Yagishita, Y., Fukutomi, T., Muramatsu, H., Negishi, T., Sugawara, A., Kensler, T.W., and Yamamoto, M. (2013). The Keap1-Nrf2 system prevents onset of diabetes mellitus. Mol. Cell. Biol. *33*, 2996–3010.

Weir, G.C., and Bonner-Weir, S. (2004). Five stages of evolving beta-cell dysfunction during progression to diabetes. Diabetes *53* (*Suppl. 3*), S16–S21.

Wiederkehr, A., Park, K.-S., Dupont, O., Demaurex, N., Pozzan, T., Cline, G.W., and Wollheim, C.B. (2009). Matrix alkalinization: a novel mitochondrial signal for sustained pancreatic β-cell activation. EMBO J. *28*, 417–428.

Willson, V.J.C., and Tipton, K.F. (1980). The effect of pH on the allosteric behaviour of ox-brain NAD^+-dependent isocitrate dehydrogenase. Eur. J. Biochem. *109*, 411–416.

Zehetner, J., Danzer, C., Collins, S., Eckhardt, K., Gerber, P.A., Ballschmieter, P., Galvanovskis, J., Shimomura, K., Ashcroft, F.M., Thorens, B., et al. (2008). PVHL is a regulator of glucose metabolism and insulin secretion in pancreatic β cells. Genes Dev. *22*, 3135–3146.

Zelent, D., Najafi, H., Odili, S., Buettger, C., Weik-Collins, H., Li, C., Doliba, N., Grimsby, J., and Matschinsky, F.M. (2005). Glucokinase and glucose homeostasis: proven concepts and new ideas. Biochem. Soc. Trans. *33*, 306–310.

Zhang, Q., Ramracheya, R., Lahmann, C., Tarasov, A., Bengtsson, M., Braha, O., Braun, M., Brereton, M., Collins, S., Galvanovskis, J., et al. (2013). Role of KATP channels in glucose-regulated glucagon secretion and impaired counterregulation in type 2 diabetes. Cell Metab. *18*, 871–882.

Zheng, L., MacKenzie, E.D., Karim, S.A., Hedley, A., Blyth, K., Kalna, G., Watson, D.G., Szlosarek, P., Frezza, C., and Gottlieb, E. (2013). Reversed argininosuccinate lyase activity in fumarate hydratase-deficient cancer cells. Cancer Metab. *1*, 12.

Zheng, L., Cardaci, S., Jerby, L., MacKenzie, E.D., Sciacovelli, M., Johnson, T.I., Gaude, E., King, A., Leach, J.D., Edrada-Ebel, R., et al. (2015). Fumarate induces redox-dependent senescence by modifying glutathione metabolism. Nat. Commun. *6*, 6001.

Cell Reports, Volume *20*

Supplemental Information

Fumarate Hydratase Deletion in Pancreatic

β Cells Leads to Progressive Diabetes

Julie Adam, Reshma Ramracheya, Margarita V. Chibalina, Nicola Ternette, Alexander Hamilton, Andrei I. Tarasov, Quan Zhang, Eduardo Rebelato, Nils J.G. Rorsman, Rafael Martín-del-Río, Amy Lewis, Gizem Özkan, Hyun Woong Do, Peter Spégel, Kaori Saitoh, Keiko Kato, Kaori Igarashi, Benedikt M. Kessler, Christopher W. Pugh, Jorge Tamarit-Rodriguez, Hindrik Mulder, Anne Clark, Norma Frizzell, Tomoyoshi Soga, Frances M. Ashcroft, Andrew Silver, Patrick J. Pollard, and Patrik Rorsman

Supplemental Index

Supplemental Experimental Procedures

Supplemental References

Supplemental Table and Figure Legends

Supplemental Figure S1. Introduction of full length (FH) and cytoplasmic (FH^{cyt}) human fumarate hydratase rescues dysregulated metabolism in Fh1βKO islets.

Supplemental Figure S2. Glucose stimulated insulin secretion is blunted in Fh1βKO islets

Supplemental Figure S3. Glucose stimulated amino acid response in CTL and Fh1βKO islets

Supplemental Figure S4. Metabolite analysis with stable isotope tracer [U-$^{13}C_5$]-glutamine or $^{13}C_6$-glucose of murine Fh1βKO islets and CTL islets.

Supplemental Figure S5. Introduction of full length (FH) or cytoplasmic (FH^{cyt}) human fumarate hydratase restores mitochondrial morphology in β-cells lacking *Fh1*.

Supplemental Figure S6. Evidence of elevated protein succination in pancreatic islets from Fh1βKO mice and human diabetic donors.

Supplemental Figure S7. Glyceraldehyde blunts GSIS in CTL islets.

Supplemental Table 1. Metabolite analysis of murine Fh1βKO islets compared to CTL islets confirms *Fh1* loss leads to dysregulated metabolism.

Supplemental Table 2. Screening protein succination in Fh1βKO murine islets, non-diabetic and T2D human islets by mass spectrometry.

Supplemental Experimental Procedures

RNA isolation, reverse transcription and quantitative PCR (qPCR)
Total RNA was extracted from freshly-picked size-matched islets using Tri-reagent (Sigma) according to the manufacturer's protocol. Single-strand cDNA was synthesized from total RNA (2 µg) using a high-capacity cDNA kit (Applied Biosystems) and analyzed by multiplex qPCR using Taqman gene expression assays on a StepOne thermocycler (Applied Biosystems). Normalization was to β-actin mRNA and relative gene expression was calculated using the ΔΔCT method. DNA corresponding to 50 ng of RNA template was used and three biological replicates, each in duplicate, were performed for each experiment. Statistical comparisons were performed using an unpaired Student's *t* test.

Mouse Islet Isolation, culture and hormone secretion
Islets were isolated by collagenase (Sigma) or liberase (Roche) digestion. Collagenase or liberase was injected into the pancreas, which was then excised and incubated for 10-14 min at 37°C. Digestion was stopped by the addition of 10 ml of ice-cold Hank's Balanced Salt (HBSS; Sigma UK) supplemented with 0.1% bovine serum albumin (BSA) (Invitrogen, UK). The islets were left to sediment by gravity and washed further in HBSS with BSA to remove the remaining exocrine tissue. Mouse islets were transferred to RPMI-1640 (Sigma, UK) supplemented with 5 mM glucose, 100 U/ml penicillin, 10 µg/ml streptomycin and 10% fetal calf serum. Isolated mouse islets were cultured in this medium at 37 ^{o}C in a humidified atmosphere (5% CO_2/95% air) for 2 hr prior to experiments.

Hormone secretion was measured from batches of 10-12 islets and was counted as an experiment (unless otherwise stated). Size-matched islets were hand-picked and washed twice in glucose-free RPMI-1640 (Sigma; supplemented with 100 U/ml penicillin, 10 µg/ml streptomycin and 10% fetal calf serum). The islets were pre-incubated for 1 hr in a humidified chamber at 37 ^{o}C (5% CO_2/95% air) in 300µl of Krebs-Ringer buffer (KRB) which contained the following (mM) 140 NaCl, 3.6 KCl, 2.6 $CaCl_2$, 0.5 $MgSO_4 7H_2O$, 0.5 NaH_2PO_4, 2 $NaHCO_3$, 5 HEPES and 2 mg/ml BSA (pH adjusted to 7.4) and 1 mM glucose. The pre-incubation buffer was discarded and the islets were incubated for a further 1 hr, as indicated. In one set of experiments (Figure 3E), hormone secretion was measured in the presence of 70 mM KCl (indicated in the text) to bypass electrical activity and investigate the amplifying effect of glucose.

An aliquot of the supernatant was collected and stored at -20°C for quantification of insulin or glucagon secretion by radioimmunoassay. The remaining supernatant was discarded and the islets were lysed in 100 µl of ice-cold acid ethanol solution (containing ethanol, H_2O and HCl in a ratio of 52:17:1) to release their hormone content. The lysates were immediately frozen at -20 ^{o}C. Radio-immunoassays for insulin and glucagon were performed using kits (Millipore and Eurodiagnostica respectively) following the manufacturer's protocols.

Insulin and glucagon content of whole pancreas was determined by freezing and then grinding the pancreas, after weighing. This ground tissue was then transferred to ice-cold acid ethanol (as per above) and sonicated prior to determination of hormone content.

Whole-pancreas perfusion
Dynamic measurements of insulin secretion were performed using *in situ* pancreatic perfusion. Briefly, the aorta was cannulated by ligating above the coeliac artery and below the superior mesenteric artery, and the pancreas was perfused with KRB solution at a rate of ~0.45 ml/min using an Ismatec Reglo Digital MS2/12 peristaltic pump. The perfusate was maintained at 37 ^{o}C with a Warner Instruments temperature control unit TC-32 4B in conjunction with a tube heater (Warner Instruments P/N 64-0102) and a Harvard Apparatus heated rodent operating table. The effluent was collected, using a Teledyne ISCO Foxy R1 fraction collector. The pancreas was first perfused for 20 min with 1 mM glucose before commencing the experiment to establish the basal rate of secretion.

Membrane potential recordings
Freshly isolated mouse islets were immobilized by using a large bore glass electrode and electrical activity was measured from β cells on the periphery of the islets using an EPC-10 amplifier (HEKA Electronics) and Pulse software (version 8.81, HEKA Electronics). β-cells were identified by Na^+ current inactivation pattern and electrical activity in response to different glucose concentrations. In order to maintain metabolically intact cells, perforated patch whole-cell configuration was used. The extracellular solution is composed of (mM) 140 NaCl, 3.6 KCl, 0.5 $MgSO_4$, 0.5 NaH_2PO_4, 1.5 $CaCl_2$, 5 $NaHCO_3$ and 10 HEPES (pH7.4 with NaOH). The intracellular solution contained (mM) 76 K_2SO_4, 10 NaCl, 10 KCl, 1 $MgCl_2$ and 5 HEPES (pH 7.35 with KOH). Perforation

was achieved by the pore-forming antibiotic amphotericin B (at the final concentration of 40 µg/ml) added into the intracellular solution.

Measurement of [5-^{3}H]-glucose utilization and [U-^{14}C]-glucose oxidation
Groups of islets were incubated in KRB containing BSA (Sigma, 0.2%) and 1 or 20 mM glucose at 37 °C for 90 min in vials. Metabolism was stopped by the addition of HCl. The CO_2 produced during oxidation was captured in 400 µl phenylethylamine (Sigma), diluted 1:1 v/v in methanol, during a 90 min incubation period. The $^{3}H_2O$ produced was left to equilibrate to 2 ml water added to the vials, for a further 24 hr. Biodegradable scintillation liquid (10 ml) was then added to either the 400 µl phenylethylamine or to the 2 ml H_2O, in a scintillation vial. The $^{14}CO_2$ and the $^{3}H_2O$ were measured in a scintillation counter. Radiolabeled glucose and scintillation liquid were obtained from Perkin Elmer.

HPLC quantification of amino acids
Islet amino acids were separated by reverse-phase HPLC after pre-column derivatization with o-phthaldialdehyde and quantified by fluorescence detection. Groups of 30 islets were analyzed in triplicate from Stage II Fh1βKO and CTL littermates. The content and release of the measured islet amino acids were studied following previously published methods (Hernandez-Fisac et al, 2006). The residual incubation medium was aspirated, the islets were washed twice with 100 µl PBS and their amino acids extracted with 30 µl of 10% (w/v) 5-sulfosalicylic acid.

Metabolite analysis (CE-TOFMS) of islets
The levels and pattern of incorporation of the respective labels in metabolites (fmol/cell) were determined by capillary electrophoresis time of flight mass spectrometry (CE-TOFMS) analysis. Isolated Islets from individual mice of the same genotype were pooled and then separated into experimental groups. These islets were then washed and incubated at 37 °C in replicates under appropriate experimental conditions. Labelling studies were performed on islets isolated from Stage II Fh1βKO and age-matched CTL mice and cultured in RPMI-1640 (Sigma, UK) medium supplemented with 100 U/ml penicillin, 10 µg/ml streptomycin and 10% fetal calf serum containing either the stable isotope tracer [U-$^{13}C_5$]-glutamine for 3 hr or $^{13}C_6$-glucose for 1 hr at 1 or 20 mM glucose. At the end of the incubation, the islets were washed twice in an excess of 5% mannitol in water (Wako) and 200 µl of methanol was added containing 3 standards (methionine sulfone, 2-morpholinoethanesulfonate and D-Camphor-10-sulfonic acid, each at 25 µM). The islets were then left to rest for 10 min prior to freezing and storage at -80 °C. Frozen islet samples were prepared and analyzed by CE-TOFMS as described previously (Soga et al, 2006; Soga et al, 2009).

Electron microscopy and comparative quantitation of mitochondria
Isolated islets from Stage II CTL and Fh1βKO mice were fixed in 2.5% glutaraldehyde (Sigma, UK) in 0.1 M phosphate buffer, post-fixed in 1% osmium tetroxide, block stained in 2% uranyl acetate and embedded in Spurr's resin or London Resin Gold resin (LRG) (Agar Scientific, UK). Ultrathin sections were cut onto nickel grids, contrast was enhanced with 2% uranyl acetate and lead citrate and sections examined in a Joel 1010 microscope. For morphometry, mitochondrial cytoplasmic density and sectional area (size) were quantified from micrographs (x800 magnification) taken of 25-30 β cells in 3-5 islets from each experimental group.

Culture of human islets and CE-TOFMS
Human pancreata (Figure 5E and Table S2) were obtained with ethical approval and clinical consent from non-diabetic donors. Islets were isolated in the Diabetes Research & Wellness Foundation Human Islet Isolation unit (Oxford, UK) using modified versions of published procedures (Lake et al., 1989). Following isolation, islets were cultured in CMRL medium containing 5.5 mM glucose and 2 mM L-glutamine. Hand-picked islets were size-matched and cultured for 1 or 24 hr in RPMI containing glucose as indicated. Islets were pre-incubated in Krebs-Ringer buffer (KRB) containing 2 mg/ml BSA and 3 mM glucose for 1 hr at 37 °C, followed by a 1 hr test incubation in KRB supplemented with glucose as indicated. At the end of the incubation time the islets were processed for CE-TOFMS as described above for mouse islets.

Culture of human islets and GC/MS analysis
For these experiments (Figure 5F), human islets were obtained from the Nordic Center For Clinical Islet Transplantation (Uppsala, Sweden). Experimental procedures were approved by the Lund University Ethical Board. These were in compliance with the Declaration of Helsinki (2000) and the World Medical Association. Islets were processed according as previously described (Fadista et al., 2014). Islets were incubated 30 min at 2.8 mM glucose and then stimulated with either 2.8 or 16.7 mM glucose for 1 hr. The islets were transferred into 300 µl ice-cold extraction solvent. Metabolite extracts were analyzed on an Agilent 6890N gas chromatograph (Agilent Technologies, Atlanta, GA) equipped with an Agilent 7683B auto-sampler (Agilent Technologies) and

coupled to a LECO Pegasus III TOFMS electron impact time-of-flight mass spectrometer (LECO Corp., St. Joseph, MI).

Immunofluorescence and immunoblotting

Immunofluorescence was performed using the same antibodies as for IHC with Alexa Fluor® secondary antibodies (Molecular Probes, Life technologies) and an FITC-conjugated anti-V5 antibody (Life technologies) using a Zeiss LSM510 META confocal imaging system.

Immunoblotting was performed as described previously (O'Flaherty et al., 2010) using primary antibodies against FH (Autogen Bioclear) and calnexin (Calbiochem).

Mass spectrometry (MS) and Proteome analysis

Isolated pancreatic islets were lysed in 7 M Urea /SDS buffer and either run on an SDS-PAGE or processed in solution. For in solution samples, depletion of serum albumin was achieved by addition of ice-cold ethanol to a final concentration of 42% to the lysates and reconstitution of protein pellets in a buffer containing 6 M urea and 100 mM Tris (pH 7.8). Unmodified cysteine residues were alkylated in reducing conditions (10 mM DTT) using 30 mM 4-vinylpyridine (resulting in pyridylethylation, PE) or 50 mM iodoacetamide (carbamidomethylation, CA). Proteins were then subjected to trypsin digestion. In solution samples were further purified on Sep-Pak C18 columns (Waters).

Analysis of resulting peptides was performed on either an LTQ Orbitrap Velos or a Q-Exactive (Thermo Fisher Scientific).

LTQ Orbitrap Velos

Peptides were separated on an Acquity nano UPLC system (Waters) supplemented with a 25 cm C18 column, 1.7 µm particle size (Waters). They were eluted by applying a 60-180 min linear gradient from 1% buffer A (0.1% formic acid in water) to 40% buffer B (0.1% formic acid in acetonitrile) at a flow rate of 250 nl/min. Collision-induced dissociation (CID) was induced on the twenty most abundant ions per full MS scan using an isolation width of 1.5Da. All fragmented precursor ions were actively excluded from repeated MS/MS analysis for 15 s.

Q-Exactive

Peptides were separated on a Ultimate 3000 RSLCnano System utilizing a PepMap C18 column, 2 µm particle size, 75 µm x 50 cm (Thermo Scientific). A 60 min linear gradient was applied from 1% buffer A (0.1% formic acid, 5% DMSO in water) to 40% buffer B (0.1% formic acid, 5% DMSO in acetonitrile) at a flow rate of 250 nl/min. Collision-induced dissociation (CID) was induced on the fifteen most abundant ions per full MS scan using an isolation width of 1.5 Da. All fragmented precursor ions were actively excluded from repeated MS/MS analysis for 15 s.

All samples were analyzed in triplicate. Feature identification was performed by generation of Mascot generic files (mgf) using Proteowizard and analysis with either Mascot v2.3.01 or Peaks 7 searching the human or mouse SwissProt database.

Normalisation of MS runs and label-free quantitation of detected features was performed using Peaks 7 (Bioinformatics solutions). All features with a significant change between both conditions (ANOVA p-value < 0.05) were included in the analysis and imported to Ingenuity.

Supplemental References

Fadista, J., Vikman, P., Laakso, E.O., Mollet, I.G., Esguerra, J.L., Taneera, J., Storm, P., Osmark, P., Ladenvall, C., Prasad, R.B., et al. (2014). Global genomic and transcriptomic analysis of human pancreatic islets reveals novel genes influencing glucose metabolism. PNAS *111*, 13924-13929.

Hernandez-Fisac, I., Fernandez-Pascual, S., Ortsater, H., Pizarro-Delgado, J., Martin del Rio, R., Bergsten, P., Tamarit-Rodriguez, J. (2006). Oxo-4-methylpentanoic acid directs the metabolism of GABA into the Krebs cycle in rat pancreatic islets. Biochem J *400*, 81-89

Lake, S.P., Bassett, P.D., Larkins, A., Revell, J., Walczak, K., Chamberlain, J., Rumford, G.M., London, N.J., Veitch, P.S., Bell, P.R. et al. (1989). Large-scale purification of human islets utilizing discontinuous albumin gradient on IBM 2991 cell separator. Diabetes *38* Suppl 1, 143-145.

O'Flaherty, L., Adam, J., Heather, L.C., Zhdanov, A.V., Chung, Y.L., Miranda, M.X., Croft, J., Olpin, S., Clarke, K., Pugh, C.W. et al. (2010). Dysregulation of hypoxia pathways in fumarate hydratase-deficient cells is independent of defective mitochondrial metabolism. Hum Mol Genet *19*, 3844-3851.

Soga, T., Baran, R., Suematsu, M., Ueno, Y., Ikeda, S., Sakurakawa, T., Kakazu, Y., Ishikawa, T., Robert, M., Nishioka, T. et al. (2006). Differential metabolomics reveals ophthalmic acid as an oxidative stress biomarker indicating hepatic glutathione consumption. J Biol Chem *281*, 16768-16776.

Soga, T., Igarashi, K., Ito, C., Mizobuchi, K., Zimmermann, H.P., Tomita, M. (2009). Metabolomic profiling of anionic metabolites by capillary electrophoresis mass spectrometry. Anal Chem *81*, 6165-6174.

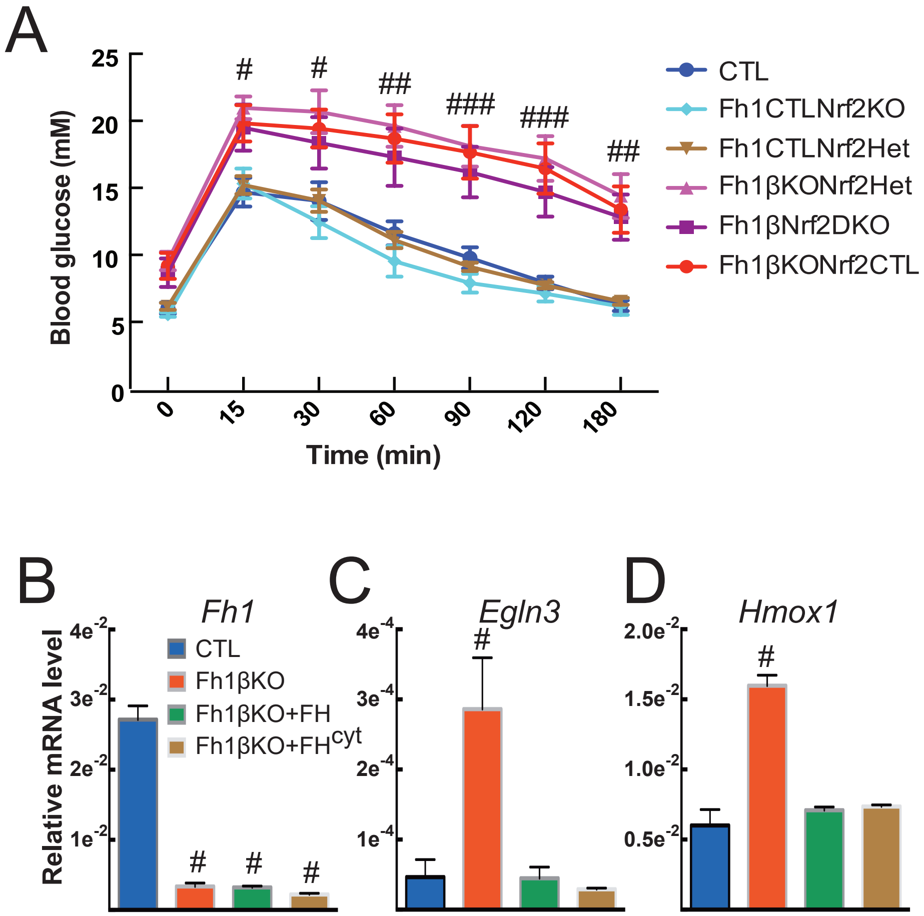

Figure S1. Introduction of full length (FH) and cytoplasmic (FHcyt) human fumarate hydratase rescues dysregulated metabolism in Fh1βKO islets. Related to Figure 1.

(**A**) Deletion of *Nrf2* did not alter the glucose tolerance in Stage II Fh1βKO compared to littermate CTL mice. IPGTT was performed in Fh1βKONrf2CTL, Fh1βKONrf2Het, Fh1βNrf2DKO and age matched CTL, Fh1CTLNrf2KO and Fh1CTLNrf2Het mice (n= 3 experiments using a total of at least 8 mice per genotype). #p<0.05, ##p<0.01 and ###p<0.001 between Fh1CTL and Fh1βKO groups irrespective of whether mice were heterozygous for *Nrf2* (Nrf2Het) or *Nrf2* was deleted (Nrf2KO).

(**B**-**D**) Analysis of gene expression by qPCR in isolated islets from Stage II CTL (blue), Fh1βKO (red), Fh1β KO+FH (green) and Fh1βKO+FHcyt (brown) mice for murine fumarate hydratase, *Fh1* (**B**), Egl-9 family hypoxia-inducible factor 3, *Egln3* (**C**), and heme oxygenase-1, *Hmox1* (**D**) (n=3 experiments using islets from at least 9 mice per genotype). These data confirm that although murine *Fh1* remains deleted in the β-cells of Fh1βKO, Fh1βKO+FH and Fh1βKO+FHcyt mice, the *Hif1α* target gene *Egln3* and the *Nrf2* target gene *Hmox1* both show mRNA expression in islets from Fh1βKO+FH and Fh1βKO+FHcyt mice comparable to that of CTL islets by virtue of expression of both full length and cytoplasmic-specific human FH. In contrast, the mRNA levels of *Egln3* and *Hmox1* are elevated in the Fh1βKO islets because of elevated fumarate leading to the subsequent stabilization of both HIF1α and NRF2. mRNA levels were normalized to those of Actb. #p<0.0001 versus CTL. Error bars indicate ± SEM.

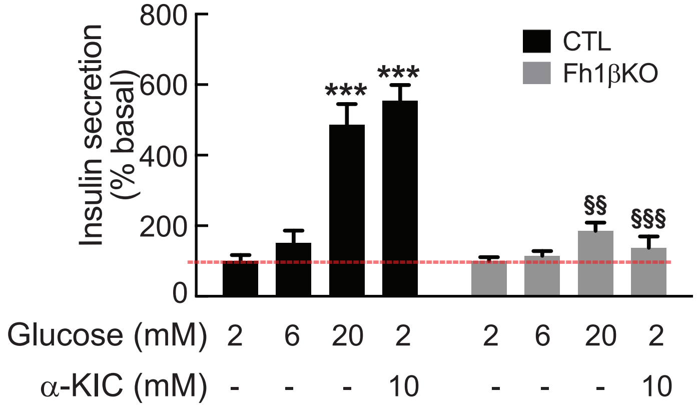

Figure S2. Glucose stimulated insulin secretion is blunted in Fh1βKO islets. Related to Figure 2.
Insulin secretion in age-matched CTL and Stage II Fh1βKO islets following 1 hr stimulation with glucose or α-ketoisocaproic acid (α-KIC; 10 mM). Results are displayed with insulin secretion expressed normalized to a percentage of the basal value (2 mM glucose). Approximate basal secretion is indicated (dashed line), (n=islets from at least 3 mice per genotype). ***$p<0.001$ versus 2 mM glucose CTL, §§$p<0.01$, §§§$p<0.001$ for comparison of the same condition between FH1βKO and CTL. Error bars indicate ± SEM.
Figures in the main text display insulin secretion data as absolute values.
Data have been normalized to the basal insulin release to compensate for any decrease in secretion due to lowered insulin content. The fact that stimulated insulin secretion remains strongly reduced in Fh1βKO islets argues that there is a functional defect.

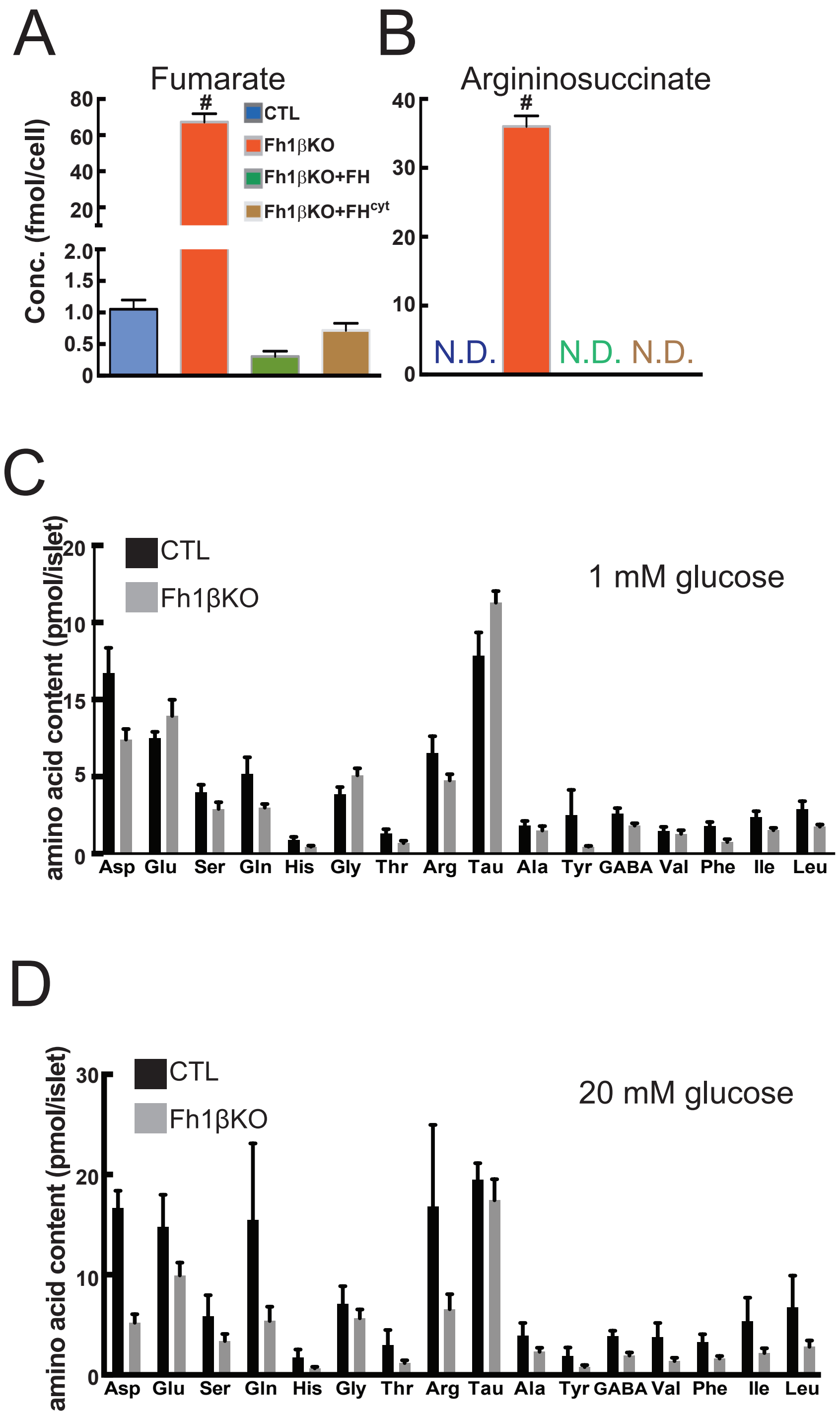

Figure S3. Glucose stimulated amino acid response in CTL and Fh1βKO islets. Related to Figure 3.
(**A-B**) Analysis by CE-TOFMS of islet content of significant metabolites from Stage II CTL (blue), Fh1βKO (red), Fh1βKO+FH (green) and Fh1βKO+FHcyt (brown) mice demonstrated that β cell fumarate (**A**) and argininosuccinate (**B**) contents were normalized to levels comparable to CTL mice following re-expression of full-length or cytoplasmic-specific human FH. #p<0.0001 versus CTL; ND not detected. (n=3 samples from 12-15 mice per experimental group).
(**C-D**) Amino acid content measured by HPLC in Stage II CTL (n=8 mice) and Fh1βKO (n=12 mice) islets after culture in 1 mM glucose (**C**) or 20 mM glucose (**D**) for 1 hr. (n=30 islets per genotype were analyzed in triplicate in 5 experiments). Abbreviations: Asp, aspartate; Glu, glutamic acid; Ser, serine; Gln, glutamine; His, histidine; Gly, glycine; Thr, threonine; Arg, arginine; Tau, taurine; Ala, alanine; Tyr, tyrosine; GABA, gamma aminobutyric acid; Val, valine; Phe, phenylalanine; Ile, isoleucine and Leu, leucine. Error bars represent ± SEM.

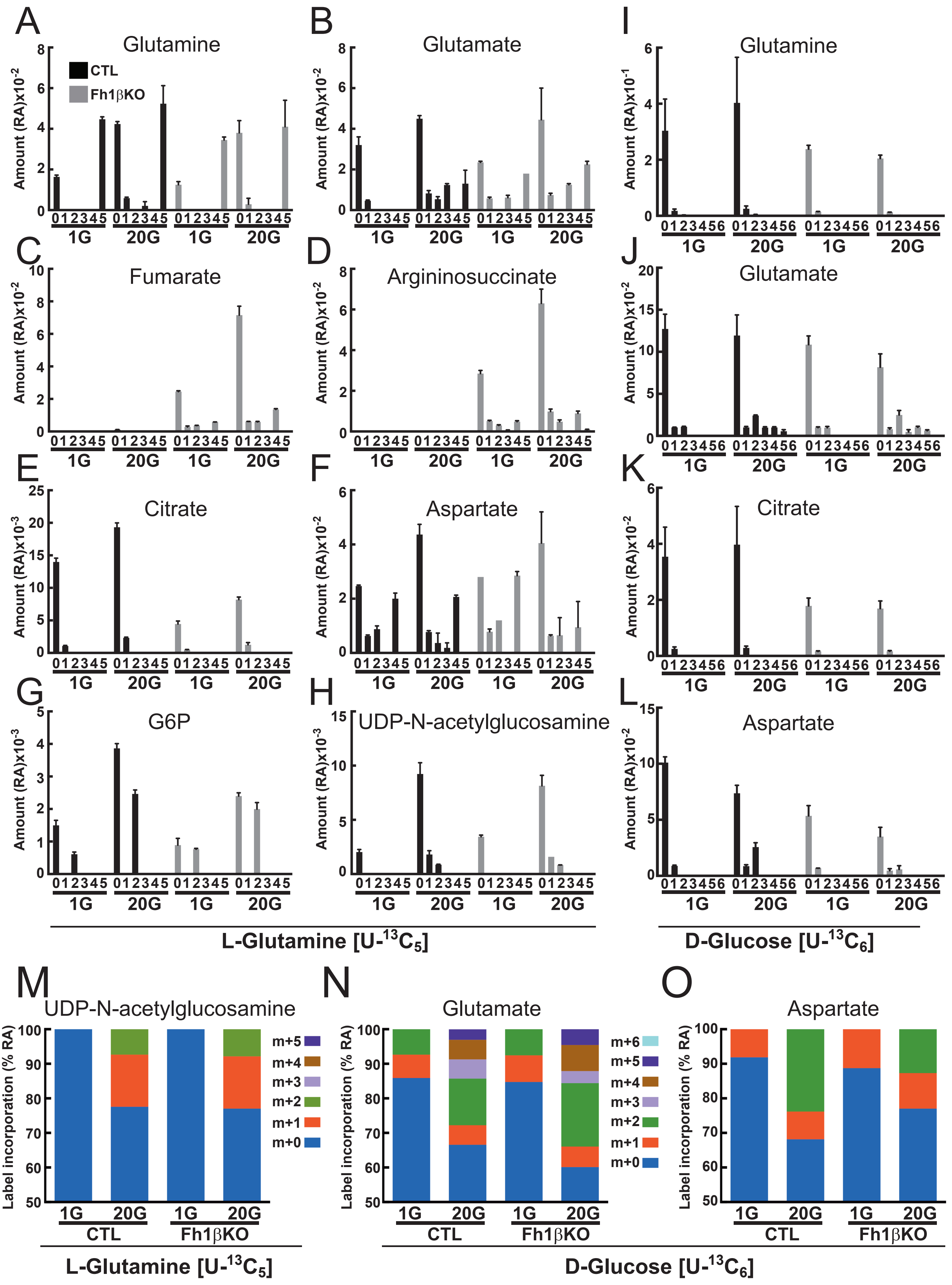
A
Glutamine
CTL
Fh1βKO
Amount (RA)x10-2
B
Glutamate
I
Glutamine
Amount (RA)x10-1
C
Fumarate
D
Argininosuccinate
J
Glutamate
E
Citrate
Amount (RA)x10-3
F
Aspartate
K
Citrate
G
G6P
H
UDP-N-acetylglucosamine
L
Aspartate
1G
20G
L-Glutamine [U-13C5]
D-Glucose [U-13C6]
M
UDP-N-acetylglucosamine
N
Glutamate
O
Aspartate
Label incorporation (% RA)
m+0
m+1
m+2
m+3
m+4
m+5
m+6
CTL
Fh1βKO

Figure S4. Metabolite analysis with stable isotope tracer [U-$^{13}C_5$]-glutamine or $^{13}C_6$-glucose of murine Fh1βKO islets and CTL islets. Related to Figure 3.

(**A-O**) Analysis by CE-TOFMS of metabolite levels (relative area) and the pattern of ^{13}C incorporation in islets isolated from age-matched CTL (black) and Stage II Fh1βKO (grey) mice following culture in medium containing either[U-$^{13}C_5$]-glutamine for 3 hr (**A-H** and **M**) or $^{13}C_6$-glucose for 1 hr (**I-L** and **N-O**) in cells and stimulation at 1 or 20 mM glucose. The samples are underivatized and uncorrected for natural abundance of ^{13}C.

(**M**) Changes in the percentage incorporation by glucose stimulation (1 and 20 mM glucose; 1G and 20G) of glutamine isotopomers (m0 to m+5) into UDP-N-acetylglucosamine following culture for 3 hr of isolated CTL and Fh1βKO islets in medium containing the stable isotope [U-$^{13}C_5$]-glutamine.

(**N**) Changes in the percentage incorporation by glucose stimulation (1 and 20 mM glucose) of glucose isotopomers (m0-m+6) into glutamate following culture for 1 hr of isolated CTL and Fh1βKO islets in medium containing the stable isotope $^{13}C_6$-glucose.

(**O**) Changes in the percentage incorporation by glucose stimulation (1 and 20 mM glucose) of glucose isotopomers (m0-m+6) into aspartate following culture for 1 hr of isolated CTL and Fh1βKO islets in medium containing the stable isotope ^{13}C-glucose.

(n=3 experimental groups of pooled islets for each experimental condition from at least 3 mice per genotype).

These data indicate that at 1 mM glucose none of the intracellular glutamate is derived from glutamine in CTL islets compared to ~50% in Fh1βKO islets. At 20 mM glucose, both the CTL and Fh1βKO islets use glutamine to produce glutamate. The small amount of islet tissue that could be isolated precluded a broad-spectrum analysis. However, at 1 mM glucose (1G) essentially none of the glutamate in the CTL islets came from labelled glutamine. By contrast, ~50% of the glutamate is derived from glutamine in Fh1βKO islets as label travels via the oxidative route of the Krebs cycle into fumarate and argininosuccinate. Most of this is from the direct conversion of glutamine to glutamate (m+5), but detection of m+3 may result from entry of fumarate into the urea cycle, allowing Asp m+4 to be produced. At 20 mM glucose, both the CTL and Fh1βKO islets use glutamine to produce glutamate, and both m+5 and m+3 are observed.

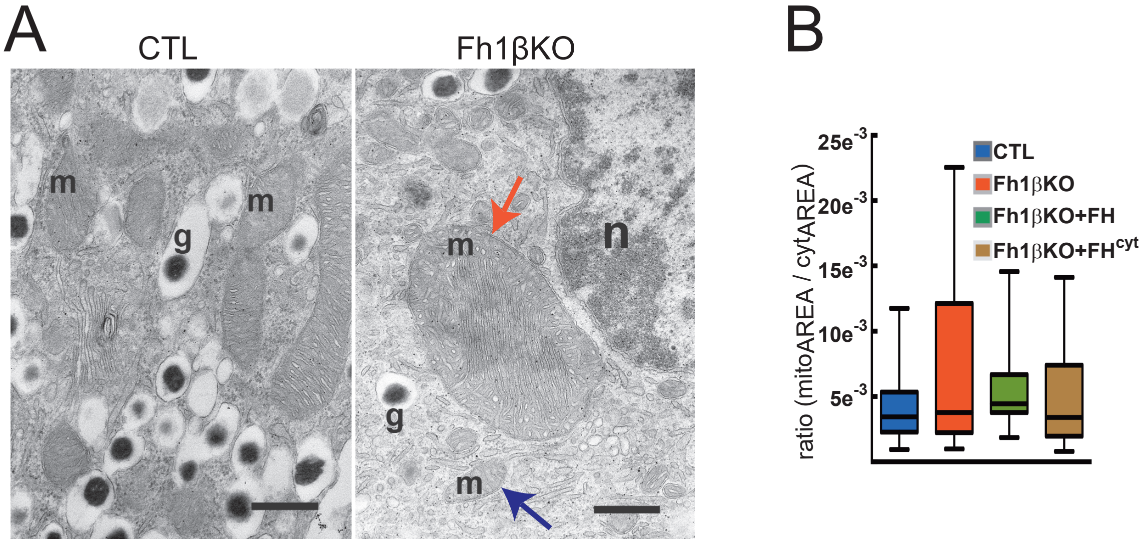

Figure S5. Introduction of full length (FH) or cytoplasmic (FHcyt) human fumarate hydratase restores mitochondrial morphology in β cells lacking *Fh1*. Related to Figure 4.

(**A**) Electron micrographs of β cells in Stage II CTL and Fh1βKO islets highlighting some very large mitochondria (red arrow) and smaller mitochondria (blue arrow) in the Fh1βKO β cells compared to CTL islets. Abbreviations: g, secretory granule; m, mitochondrion; n, nucleus. Scale bar: 500 nm.

(**B**) Relative mitochondrial area (mitochondrial area divided by cytoplasmic area; maximum to minimum range) compared in β cells in Stage II CTL (blue), Fh1βKO (red), Fh1βKO+FH (green) and Fh1βKO+FHcyt (brown) mice showing the larger range of mitochondrial areas in Fh1βKO islets and normalization following reintroduction of FH. (n=25-30 β cells in 3-5 islets per genotype). Error bars represent ± SEM.

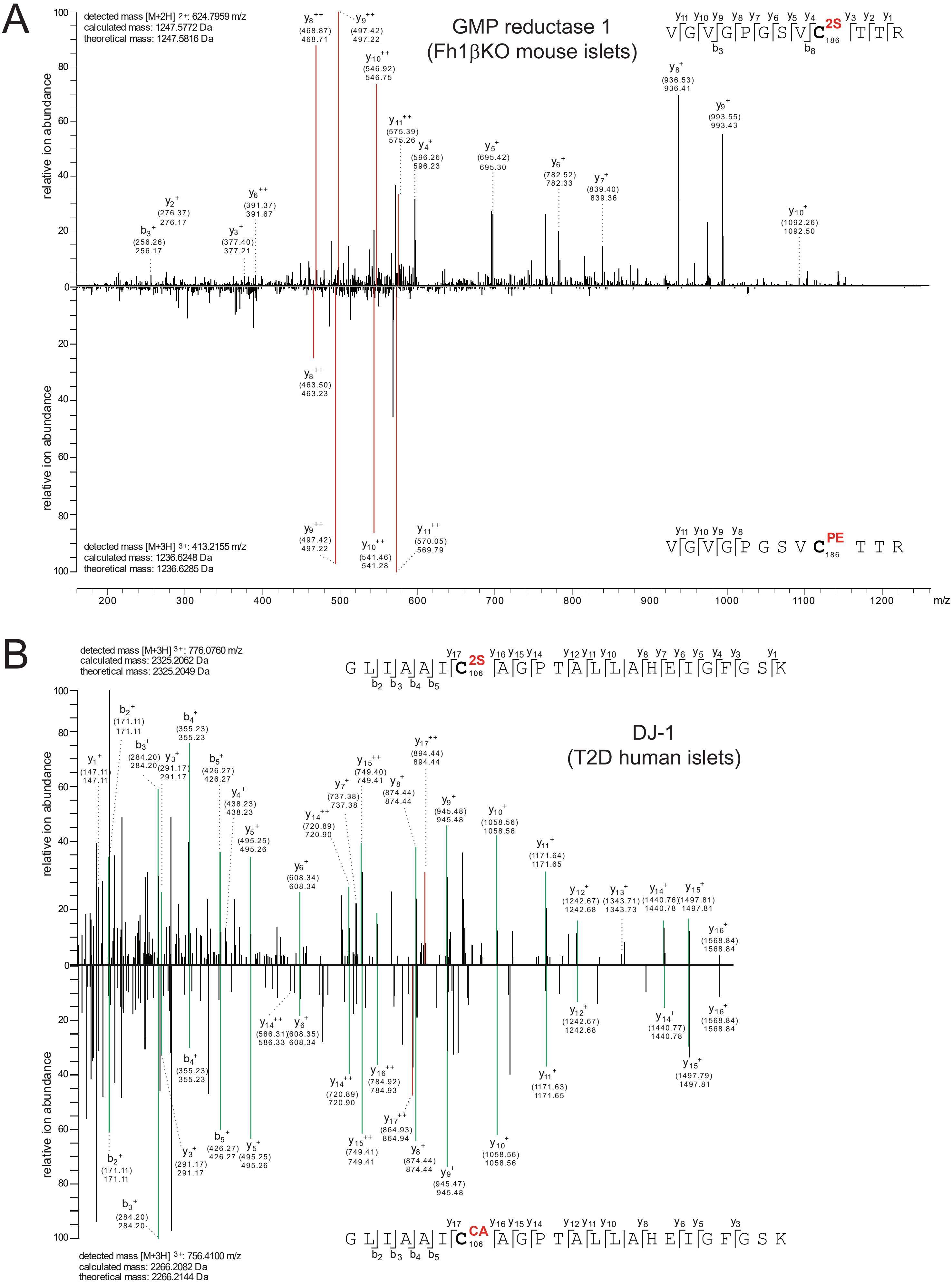
A
detected mass [M+2H] 2+: 624.7959 m/z
calculated mass: 1247.5772 Da
theoretical mass: 1247.5816 Da
GMP reductase 1
(Fh1βKO mouse islets)
V G V G P G S V C 2S 186 T T R
detected mass [M+3H] 3+: 413.2155 m/z
calculated mass: 1236.6248 Da
theoretical mass: 1236.6285 Da
V G V G P G S V C PE 186 T T R
relative ion abundance
m/z
B
detected mass [M+3H] 3+: 776.0760 m/z
calculated mass: 2325.2062 Da
theoretical mass: 2325.2049 Da
G L I A A I C 2S 106 A G P T A L L A H E I G F G S K
DJ-1
(T2D human islets)
G L I A A I C CA 106 A G P T A L L A H E I G F G S K
detected mass [M+3H] 3+: 756.4100 m/z
calculated mass: 2266.2082 Da
theoretical mass: 2266.2144 Da
relative ion abundance
m/z

Figure S6. Evidence of elevated protein succination in pancreatic islets from Fh1βKO mice and human diabetic donors. Related to Figure 5.

(**A**) MS/MS spectra for peptide $_{178}$VGVGPGSVCTTR$_{189}$ showing increased succination in guanosine 5′-monophosphate oxidoreductase (GMP reductase 1) at cysteine 186 (C186) in Stage II Fh1βKO islets. Both theoretical mass and detected mass (in brackets) are given for each assigned fragment ion. Peptide fragments of different mass that contain the modified residue are highlighted in red. PE indicates the modification of cysteine residues at C186 to pyridylethyl-cysteine.

(**B**) MS/MS spectra showing either succination (^{2S}C) or carbamidoethylation (^{CA}C) at cysteine 106 (C106) in the $_{100}$GLIAAICAGPTALLAHEIGFGSK$_{122}$ peptide of human DJ-1 derived from islets of a T2D donor. Matching fragment ion peaks between the two peptide species that do not contain the modified residue are highlighted in green, whereas peptide fragments of different mass that contain the modified residue are highlighted in red.

The calculated peptide mass based on the detected m/z (m: mass, z: charge) value of the doubly charged precursor peptide ion ($[M+2H]^{++}$) and the calculated ([M] calc.) and theoretical peptide mass ([M] theor.) are stated for both peptide species. Detected N- and C-terminal fragment ions are indicated in the peptide sequence, assigned in the spectrum and depicted as follows: b: N-terminal fragment ion; y: C-terminal fragment ion; *: fragment ion minus NH_3; and $^{++}$: doubly charged fragment ion. Both theoretical mass (in brackets) and detected mass are given for each assigned fragment ion.

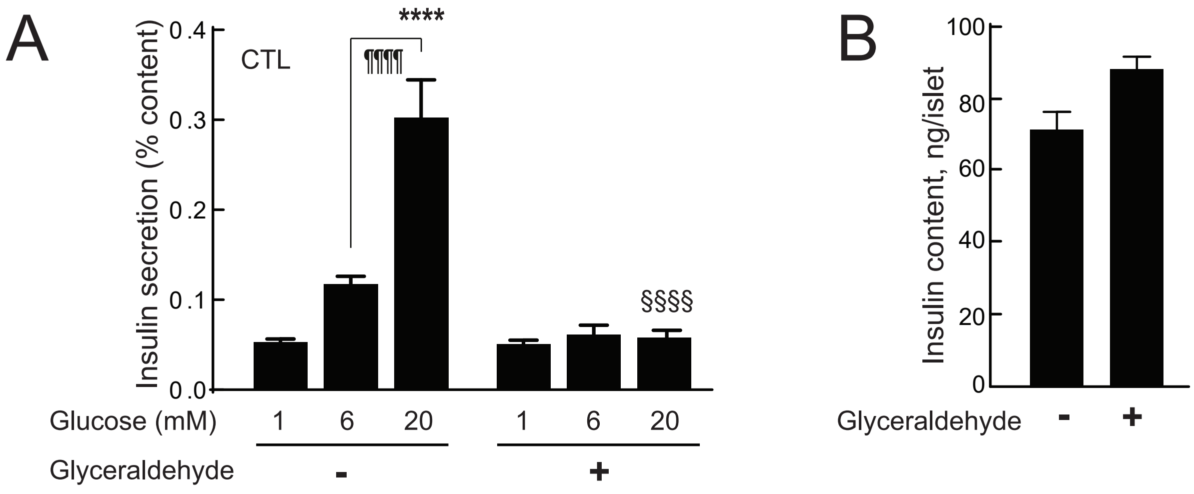

Figure S7. Glyceraldehyde blunts GSIS in CTL islets. Related to Figures 5 and 6.
(**A**) Insulin secretion measured during 1 hr incubations at 1, 6 and 20 mM glucose following 24 hr culture of wild-type islets from C57BL/6J mice with 10 mM glyceraldehyde. (n=6 experimental groups of islets for each condition assayed in 2 experiments; islets harvested from 6 mice in total). Data are mean values ± SEM of secretion expressed as per cent of hormone content. ****$p<0.0001$ versus 1 mM glucose; ¶¶¶¶$p<0.0001$ versus 6 mM glucose; §§§§$p<0.0001$ comparing same condition between groups.
(**B**) Insulin content of the islets in A.

Metabolite	WT	WT	WT	WT	WT	WT	WT	WT	KO	KO	KO	KO	KO	KO	KO	T-test
**Argininosuccinate**	0.00E+00	0.00E+00	0.00E+00	0.00E+00	0.00E+00	0.00E+00	0.00E+00	0.00E+00	9.19E-01	8.75E-01	9.39E-01	9.58E-01	9.13E-01	9.57E-01	8.28E-01	**8.72E-17**
**Fumarate**	6.53E-02	7.15E-02	1.22E-01	6.28E-02	6.81E-02	1.34E-01	7.82E-02	5.63E-02	1.20E+00	1.05E+00	1.06E+00	1.15E+00	1.21E+00	1.25E+00	1.16E+00	**1.45E-14**
**Glucose-6-phosphate**	2.46E-02	5.92E-02	4.63E-02	3.91E-02	3.26E-02	3.90E-02	3.86E-02	3.21E-02	0.00E+00	0.00E+00	0.00E+00	0.00E+00	0.00E+00	0.00E+00	3.39E-02	**7.42E-05**
**Aspartate**	1.26E+00	1.41E+00	1.54E+00	1.31E+00	1.19E+00	1.41E+00	1.33E+00	1.17E+00	1.06E+00	8.90E-01	8.96E-01	1.09E+00	1.09E+00	1.02E+00	1.08E+00	**1.09E-04**
**Adenylosuccinate**	0.00E+00	0.00E+00	0.00E+00	0.00E+00	0.00E+00	0.00E+00	0.00E+00	0.00E+00	7.07E-02	6.39E-02	5.89E-02	1.99E-01	7.08E-02	8.88E-02	1.44E-01	**1.27E-04**
**Cysteine-glutathione disulphide -Divalent**	8.49E-02	1.02E-01	1.09E-01	6.87E-02	3.75E-02	9.96E-02	6.01E-02	9.32E-02	6.97E-02	5.69E-02	0.00E+00	1.80E-02	4.40E-02	2.47E-02	1.84E-02	**2.07E-03**
**Adenosine monophosphate**	3.85E-01	5.45E-01	5.70E-01	2.40E-01	3.68E-01	6.06E-01	4.10E-01	2.74E-01	1.13E-01	1.80E-01	1.26E-01	3.26E-01	2.23E-01	9.76E-02	2.97E-01	**2.29E-03**
**Cystathionine**	2.02E-01	2.18E-01	2.79E-01	1.91E-01	1.85E-01	2.90E-01	1.83E-01	3.62E-01	3.95E-01	3.60E-01	3.85E-01	3.51E-01	3.18E-01	2.70E-01	3.19E-01	**3.37E-03**
**Uridine monophosphate**	9.10E-02	1.35E-01	1.31E-01	6.77E-02	1.15E-01	1.75E-01	1.24E-01	8.39E-02	0.00E+00	0.00E+00	0.00E+00	1.06E-01	7.35E-02	3.50E-02	1.05E-01	**6.30E-03**
**Guanidinoacetate**	5.13E-02	1.23E-01	1.22E-01	6.01E-02	5.36E-02	9.18E-02	1.02E-01	9.68E-02	5.64E-02	4.67E-02	0.00E+00	6.31E-02	5.47E-02	5.22E-02	5.48E-02	**9.36E-03**
**Gamma aminobutyricacid**	5.33E-01	7.11E-01	8.16E-01	7.00E-01	5.59E-01	7.92E-01	8.52E-01	6.80E-01	5.38E-01	4.27E-01	5.24E-01	5.85E-01	6.51E-01	5.04E-01	6.46E-01	**1.21E-02**
**Proline**	4.20E-01	4.94E-01	6.34E-01	5.39E-01	3.69E-01	5.79E-01	5.25E-01	5.01E-01	5.25E-01	3.40E-01	3.57E-01	3.85E-01	4.12E-01	3.78E-01	3.84E-01	**1.32E-02**

Metabolite	WT	WT	WT	WT	WT	WT	WT	WT	KO	KO	KO	KO	KO	KO	KO	T-test
**cis-Aconitate**	3.12E-01	2.00E-01	8.11E-02	1.50E-01	1.14E-01	1.16E-01	1.74E-01	2.73E-01	2.48E-01	4.36E-01	6.46E-01	2.32E-01	3.24E-01	2.47E-01	2.88E-01	**1.61E-02**
**Guanosine monophosphate**	1.63E-01	2.64E-01	2.72E-01	1.30E-01	1.47E-01	3.75E-01	1.99E-01	1.62E-01	0.00E+00	1.29E-01	0.00E+00	2.25E-01	1.01E-01	0.00E+00	1.77E-01	**1.78E-02**
**Glycine**	7.74E-01	8.17E-01	1.13E+00	9.82E-01	7.38E-01	9.04E-01	9.38E-01	7.81E-01	1.11E+00	9.61E-01	1.05E+00	9.93E-01	1.08E+00	9.74E-01	9.92E-01	**2.21E-02**
**Glucose 1-phosphate**	9.78E-02	1.16E-01	1.47E-01	1.05E-01	5.91E-02	9.12E-02	9.92E-02	9.52E-02	8.20E-02	7.47E-02	6.40E-02	4.43E-02	1.01E-01	9.05E-02	5.39E-02	**3.03E-02**
**Isocitrate**	6.27E-01	4.60E-01	1.57E-01	4.03E-01	2.00E-01	1.47E-01	3.55E-01	4.76E-01	4.75E-01	7.13E-01	1.09E+00	4.79E-01	5.49E-01	4.15E-01	5.01E-01	**3.35E-02**
**Glycero-phosphorylcholine**	4.44E-01	5.25E-01	6.75E-01	6.77E-01	3.99E-01	6.25E-01	7.73E-01	6.96E-01	5.94E-01	5.88E-01	7.59E-01	8.09E-01	7.48E-01	9.04E-01	9.57E-01	**3.70E-02**

Table S1. Metabolite analysis of murine Fh1βKO islets compared to CTL islets confirms *Fh1* loss leads to dysregulated metabolism. Related to Figure 3.
Analysis by CE-TOFMS of islet cellular concentrations (fmol/cell) for metabolites. Islets were isolated from individual Stage II Fh1βKO (n=7) and age-matched CTL (n=8) mice and incubated for 1 hr in 5 mM glucose then prepared for CE-TOFMS as described above. Metabolites listed show significance of $p<0.05$ or better between CTL and Fh1βKO islets (Student's *t*-test).

Table S2. Screening protein succination in Fh1βKO murine islets, non-diabetic and T2D human islets by mass spectrometry. Related to Figure 5.

This list of proteins was established using a false discovery rate of 1%. Protein sequence was confirmed on http://www.uniprot.org/. Quantitative analysis of protein changes was determined using Peaks (Bioinformatics solutions) and the proteins that were detected with significant change of abundance between both conditions are listed. P is the probability that the match between the MS/MS query and the peptide sequence is random. (n=150 islets isolated from 3 Stage II Fh1βKO mice; approximately 150 islets from each of 2 diabetic and 2 nondiabetic adult donors).
